# Sulfated Phenolic Substances: Preparation and Optimized HPLC Analysis

**DOI:** 10.3390/ijms23105743

**Published:** 2022-05-20

**Authors:** Lucie Petrásková, Kristýna Káňová, Katerina Brodsky, Anastasiia Hetman, Barbora Petránková, Helena Pelantová, Vladimír Křen, Kateřina Valentová

**Affiliations:** 1 Institute of Microbiology of the Czech Academy of Sciences, Vídeňská 1083, 142 20 Prague, Czech Republic; petraskova@biomed.cas.cz (L.P.); astriik@gmail.com (K.K.); katerina.brodsky@biomed.cas.cz (K.B.); hetmananastasiia@seznam.cz (A.H.); barbora.petrankova@natur.cuni.cz (B.P.); pelantova@biomed.cas.cz (H.P.); kren@biomed.cas.cz (V.K.); 2 Department of Biochemistry and Microbiology, University of Chemistry and Technology Prague, Technická 3, 166 28 Prague, Czech Republic; 3 Department of Pharmacology and Toxicology, Faculty of Pharmacy in Hradec Králové, Charles University, Heyrovského 1203, 500 05 Hradec Králové, Czech Republic; 4 Department of Analytical Chemistry, Faculty of Science, Charles University, Albertov 6, 128 43 Prague, Czech Republic

**Keywords:** aryl sulfotransferase, *Desulfitobacterium hafniense*, HPLC analysis, sulfates, flavonoids, polyphenols, phenolic acid

## Abstract

Sulfation is an important reaction in nature, and sulfated phenolic compounds are of interest as standards of mammalian phase II metabolites or pro-drugs. Such standards can be prepared using chemoenzymatic methods with aryl sulfotransferases. The aim of the present work was to obtain a large library of sulfated phenols, phenolic acids, flavonoids, and flavonolignans and optimize their HPLC (high performance liquid chromatography) analysis. Four new sulfates of 2,3,4-trihydroxybenzoic acid, catechol, 4-methylcatechol, and phloroglucinol were prepared and fully characterized using MS (mass spectrometry), ^1^H, and ^13^C NMR. The separation was investigated using HPLC with PDA (photodiode-array) detection and a total of 38 standards of phenolics and their sulfates. Different stationary (monolithic C18, C18 Polar, pentafluorophenyl, ZICpHILIC) and mobile phases with or without ammonium acetate buffer were compared. The separation results were strongly dependent on the pH and buffer capacity of the mobile phase. The developed robust HPLC method is suitable for the separation of enzymatic sulfation reaction mixtures of flavonoids, flavonolignans, 2,3-dehydroflavonolignans, phenolic acids, and phenols with PDA detection. Moreover, the method is directly applicable in conjunction with mass detection due to the low flow rate and the absence of phosphate buffer and/or ion-pairing reagents in the mobile phase.

## 1. Introduction

Plant constituents such as flavonoids and phenolic acids are an essential part of a so-called healthy diet. These polyphenolic substances, once considered antioxidants and protective agents against ROS, act more at the receptor level [1]. However, their function is still not fully understood, and it is necessary to thoroughly study their toxicology, metabolism, and possible interactions with dietary supplements and drugs.

Flavonoids and phenolic acids are preferentially metabolized (sulfated, methylated, or glucuronidated) via the II biotransformation phase [2,3]. Sulfated flavonoids and other polyphenols are therefore of great pharmacological interest. They can serve as potential (pro)drugs and have antiviral, antitumor, anticoagulant, and anti-inflammatory activities [4]. Sulfation is also associated with molecular recognition, cell signaling, and hormone regulation [5]. A detailed study of all these properties requires substantial amounts, which usually cannot be isolated from the biological material. On the other hand, nature-inspired sulfated conjugates can be synthesized chemically or enzymatically to provide well-characterized standards.

The enzymatic sulfation of (poly)phenolic compounds has recently been preferred to the chemical one [6,7,8,9,10,11,12,13]. Bacterial aryl sulfotransferases such as the one from *Desulfitobacterium hafniense* use *p*-nitrophenyl sulfate (*p*-NP-S) as a sulfate donor. When monitoring the reaction progress, we solve the analytical separation of polar (phenols, *p*-nitrophenol, *p*-NP) and highly polar substances (phenolic sulfates, *p*-NP-S). Besides TLC, the HPLC method is the analytical method of the first choice for monitoring sulfation reactions due to its robustness and general availability. Sulfates of various polyphenols carrying one or more highly charged sulfate group(s) are inherently very polar compounds. If the sulfate group is not modified, or if the effect of the modification on the overall polarity is small, conventional reversed-phase liquid chromatography will not provide adequate retention. This shows up on the chromatogram as peak fronting, tailing, or very broad peaks.

One way to solve this problem is to modify the sulfate group using ion-pairing liquid chromatography (IPLC). IPLC uses a conventional reversed stationary phase in combination with a mobile phase enriched in an ion-pairing reagent. The ion-pairing reagent (generally an amine) forms a hydrophobic pair with the highly charged molecule of opposite charge. This dramatically increases the retention of the analytes and thus the sharpness of the peaks. IPLC has been used, for example, for the analysis of oligonucleotides [14], for the separation of reaction mixtures with sulfates of 2-phenylethyl alcohol, *p*-NP-glycerol, *p*-NP-glucose, phenyl-glucose, *p*-NP-*N*-acetyl-D-glucosamine (GlcNAc) [6], and for 3-sulfo-17-beta-estradiol-4,4′-disulfate, and for monitoring the sulfation of resveratrol and phloretin [7,15]. However, IPLC applications may also have disadvantages, such as artifacts when using gradient elution, incompatibility with mass spectrometry (MS) and preparative chromatography, or more complex mobile phase preparations.

Therefore, in some cases, a better solution is required. Resolution and sensitivity can be greatly improved by a careful combination of stationary and mobile phases, flow rate, temperature, and the type of detection. Hereunder we show the advantages and disadvantages of analytical methods developed for the separation of sulfated substances.

C18 columns in combination with phosphate-containing mobile phases and an ion-pairing reagent required over 20 min for the separation of indoxyl sulfate, sulfated primary aliphatic alcohols, estradiol, or bisphenol-A [6,7,16]. Significantly shorter times (2.5–6 min) were reached when separating silybin or isosilybin sulfates. However, the width of the peaks in 5% of their height was greater than one minute (1–4.5 min) in all cases [9]. Polyamine II column with a linear gradient of phosphate buffers was used to separate fluorescently labeled *N*-sulfated polysaccharides [17]. However, phosphate is incompatible with the LC-MS interface.

For monitoring the metabolic fate of (poly)phenolic substances in complex experiments on animals, MS/MS detection can preferably be used. However, these devices are expensive and not generally available. The sulfated metabolites of naringin and hesperidin were monitored in in vivo experiments using the UHPLC-ESI-MS/MS (ultra-high performance liquid chromatography coupled with electrospray ionization tandem mass spectrometry) analysis (Waters Acquity BEH C18 column, gradient elution). The metabolites were identified based on chemical composition, retention time, MS/MS fragmentation pattern, and comparison with available standards and references [2]. The sulfated product of chlorocatechol was identified using the NUCLEODUR HILIC (hydrophilic interaction chromatography) column with an ion trap mass analyzer. The peak of chlorocatechol sulfate was broad and the other components of the reaction mixture (*p*-NP, 3-chlorocatechol) were not separated from the baseline [18]. The biotransformation products of quercetin, isoquercitrin, and taxifolin sulfates formed by HepG2 cells were monitored by UHPLC (XDB phenyl column) and ESI MS/MS detection [19]. The sulfation reaction (catalyzed by aryl sulfotransferase from *Clostridium innocuum*) of various phenols, flavonoids, quinones, primary alcohols, and sugars was analyzed using a C18 column (mobile phase acetonitrile, water, trifluoroacetic acid) and the identity of the reaction products was confirmed by MS [20].

The analyses of hydroxytyrosol and acetyltyrosol sulfates [21], luteolin, myricetin, and ampelopsin sulfates [8], quercetin mono- and disulfates [12] or quercetin, isoquercitrin, and taxifolin sulfates [11] on the pentafluorophenyl column (mobile phase water, methanol, trifluoroacetic acid) suffered from tailing peaks. Tailing peaks were also observed during the separation of sulfated polysaccharides (cellobiose and GlcNAc-linker-tBoc) on the Multospher APS-HP-5 µm HILIC column (mobile phase acetonitrile, ammonium acetate) [22]. HPLC chromatograms of 2,3-dehydrosilybin sulfation obtained by separation on a monolithic Chromolith C18 column (mobile phase acetonitrile, water, formic acid) also showed tailing [13].

Separation of a chemically prepared mixture of mono- and disulfates of quercetin on a BDS -Hypersil C18 column (mobile phase ammonium acetate with methanol) gave good analytical separation (sharp peaks, baseline separation); up to 20 min was achieved in the separation [23]. The sulfation reagent sulfur trioxide *N*-triethylamine probably also served as an ion-pairing reagent and allowed sharp peaks. Analyses of the sulfates of quercetin and epicatechin on the AQUA reversed-phase C18 column and the Spherisorb S3OD-2 C18 column (mobile phase trifluoroacetic acid in water and methanol, respectively) took over 30 min [24].

The following parameters can be considered as disadvantages for the separation of sulfated polyphenols: Phosphate in the mobile phase, use of ion-pairing reagent (both incompatible with LC-MS), MS/MS detection (not a common detector), separation time over 20 min, and tailing and/or coelution of the peaks (poorer separation of the peaks in the reaction mixture). The disadvantages of the published methods with at least one undesirable parameter are summarized in Table 1.

The aim of this work was to obtain a large library of sulfated phenols, phenolic acids, flavonoids, and (2,3-dehydro)flavonolignans and to develop a robust and reliable HPLC analytical method suitable for the separation of enzymatic sulfation reaction mixtures of these phenolics. The authentic standards of sulfates of 2,3,4-trihydroxybenzoic acid, catechol, 4-methylcatechol, and phloroglucinol were prepared in the frame of this work. Four types of stationary phases (pentafluorophenyl (PFP), C18, C18 Polar, and HILIC) were compared using mobile phases without or with buffer and with PDA detection. The optimal method is also suitable for mass detection because it does not use phosphate buffers and ion-paired reagents in the mobile phase. The duration of separation was up to 20 min in most cases without peak tailing and coelution.

## 2. Results and Discussion

### 2.1. Synthesis of Sulfated Phenolics

**Figure 1:**
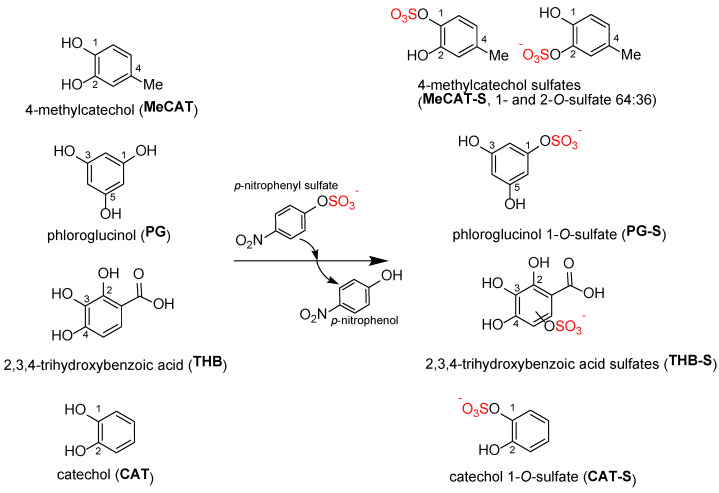
Sulfation of 4-methylcatechol (MeCAT), phloroglucinol (PG), 2,3,4-trihydroxybenzoic acid (THB), and catechol (CAT) using aryl sulfotransferase from *Desulfitobacterium hafniense* and *p*-nitrophenyl sulfate as sulfate donor.

A large number of metabolic standards used in this work were prepared earlier using the bacterial aryl sulfotransferase of *Desulfitobacterium hafniense* [8,10,11,12,13,19]. Besides previously published products, new authentic standards were prepared in the present work with the same very effective enzymatic method for selective sulfation of polyphenols (Figure 1) and fully characterized with HPLC, MS, and NMR (see Supplementary Material).

**Figure 2:**
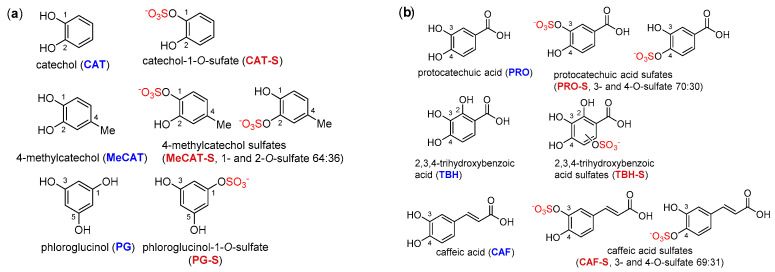
Structures of simple phenols (**a**) and phenolic acids (**b**, both blue) and their sulfates (crimson) used in this study. The sulfate groups are highlighted in red.

**Figure 3:**
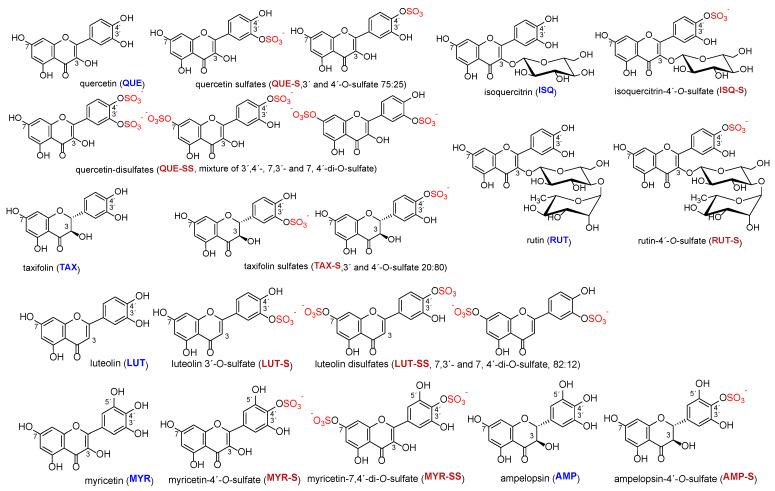
Structures of flavonoids (blue) and their sulfates (crimson) used in this study. The sulfate groups are highlighted in red.

**Figure 4:**
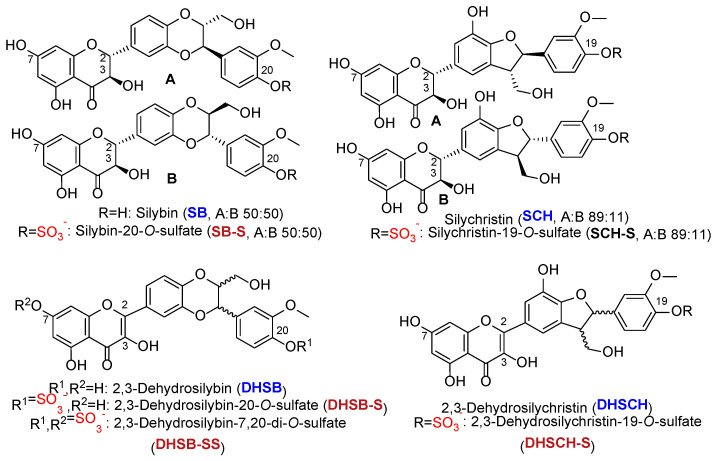
Structures of flavonolignans (blue) and their sulfates (crimson) used in this study. The sulfate groups are highlighted in red.

The structures of all phenolic compounds and their respective sulfated derivatives used in the present study are shown in Figure 2, Figure 3 and Figure 4. Altogether, these compounds form a unique library of sulfated simple phenols (Figure 2a), phenolic acids (Figure 2b), flavonoids (Figure 3), and flavonolignans (Figure 4).

#### 2.1.1. Sulfation of 4-Methylcatechol (MeCAT)

Sulfation of 4-methylcatechol yielded a product (MeCAT-S) that appeared as one spot on TLC and one peak on HPLC using Method M2. However, NMR analysis revealed a mixture of two isomers: 4-methylcatechol-1-*O*-sulfate and 4-methylcatechol-2-*O*-sulfate in a ratio of 64:36 (Table S8a,b). The reaction (Figure 1) was rapid, with the entire amount of MeCAT-S synthesized within the first 20 min. After purification by gel chromatography, 180 mg of the product was obtained from 200 mg of starting material (isolated yield 58 mol%) as a white powder with an overall purity of 99% (HPLC, Figure S1) and corresponding *m/z* (Figure S2).

#### 2.1.2. Sulfation of Phloroglucinol (PG)

In the case of PG, the formation of at least two products was observed (TLC, HPLC-Method M2, Figure 1), but only one of them was successfully isolated and characterized. According to NMR and MS analyses, it was confirmed to be a monosulfate. The product was isolated as a brownish powder (73 mg, 44% yield, 90% purity, Figures S11 and S12, Table S13).

#### 2.1.3. Sulfation of Protocatechuic Acid (PRO)

When PRO was sulfated, only one sulfated product was observed (TLC, HPLC-Method M2). According to the NMR analysis, it was a mixture of two regioisomers: protocatechuic acid-3-*O*-sulfate and protocatechuic acid-4-*O*-sulfate in the ratio of 7:3. The product tended to associate with solvents and other chemicals used during the purification process (H_2_O, MeOH, HCOOH); however, after repeated purification, it was isolated as a white powder (12 mg, 4% yield, 99% HPLC purity, Figure S3, Tables S9a,b).

#### 2.1.4. Sulfation of 2,3,4-Trihydroxybenzoic Acid (THB)

The gradual formation of one product was observed (TLC, HPLC-Method M2, Figure 1). Furthermore, this product tended to associate with solvents. The purification on gel chromatography gave a brown oil product (34 mg, 8% yield, 96% HPLC purity, Figures S5 and S6, Tables S10a,b).

#### 2.1.5. Sulfation of Caffeic Acid (CAF)

The sulfation of caffeic acid yielded a product that behaved as one spot on TLC and one peak on HPLC. Again, NMR analysis revealed a mixture of 3- and 4-*O*-sulfate in the ratio of 69:31. After purification, we obtained 301 mg of white-brownish oily solid (100% yield, 99% HPLC purity, Figures S7 and S8, Table S11a,b).

#### 2.1.6. Sulfation of Catechol (CAT)

The sulfation of catechol (Figure 1) led to surprisingly only one regioisomer, catechol-1-*O*-sulfate, as confirmed by MS and NMR. After purification, 116 mg of the product was obtained (34% yield, 98% HPLC purity, Figures S9 and S10, Table S12) as a white powder.

### 2.2. Development and Optimization of Analytical Methods for the Separation of Sulfated Polyphenols

The typical composition of the reaction mixture to be analyzed was as follows: *p*-NP, *p*-NP-S, the parent compound, and its sulfate (see Figure 1; in some cases, isomers and/or disulfate(s) were also present). The main focus was on monitoring the separation of all components in the mixture, the duration of the separation, and the width of the peaks at five percent of their height. The following limits were established for evaluating the width of the peaks: w_0.05_ < 0.300, very good; 0.300 < w_0.05_ < 0.500, good; and w_0.05_ > 0.500, poor.

### 2.3. Separations without Buffer in the Mobile Phase

Based on our previous experience and the articles published so far (Table 1), we decided to test these four columns: Kinetex PFP, ZICpHILIC, Chromolith RP 18e, and Luna Omega Polar C18. Initially, the Kinetex PFP, Chromolith RP 18e, and Luna Omega Polar C18 columns were each tested without the use of buffers in the mobile phase (Table 2). The use of buffers in the mobile phase is always associated with an increased washing effort of the analytical system and thus a higher time requirement, so we tried to avoid this in the first step. Since the samples contain ionizable compounds, formic acid or TFA (0.1%) was added to all mobile phases to achieve a pH of about 3. The non-ionized form is less polar and therefore more strongly retained in a reverse phase system.

The pentafluorophenyl column offers unique polar and aromatic selectivity thanks to the fluorine atoms at the periphery of the phenyl units anchored to the core-sell silica support. The Kinetex PFP column was tested using Method M3 (0.1% TFA, MeOH as mobile phases) for the separation of quercetin (QUE), isoquercitrin (ISQ), rutin (RUT), taxifolin (TAX), luteolin (LUT), myricetin (MYR), and ampelopsin (AMP) and their sulfates. Although the pH of the mobile phase was very low (pH = 2.0, column limit is 1.5) and the ionization of compounds (sulfates and their parent compounds) should be suppressed, the separation was not satisfactory in many cases. The width of the peak in the 0.5% of its height (w_0.05_) ranged from 0.295 to 1.703 (Table 3). The widest peaks were observed in quercetin and quercetin sulfates (w_0.05_ 0.554–1.703), luteolin sulfates (w_0.05_ 0.655–1.703), and myricetin sulfates (w_0.05_ 0.542–1.398). The values of w_0.05_ for the other tested substances were up to 0.500. No coelution of peaks was observed in the reaction mixtures. The longest retention time was 20.010 min (luteolin), but for most of the analyzed substances, it ranged from 6 to 16 min.

We also tested two C18 columns, one monolithic (Chromolith RP18e, Method M6), and one specially designed for increased retention of polar compounds (Luna Omega Polar C18, Method M7, Table 2). The mobile phase was the same in both cases (acetonitrile 5% and 80%, water, 0.1% formic acid, pH 2.7; the pH limits of the columns are 2.0 and 1.5, respectively). The Luna Omega Polar C18 stationary phase is a combination of a universal C18 ligand and a polar modified surface that provides improved polar retention and aqueous stability. Due to the smaller particle size of the stationary phase (3 µm) and the associated higher backpressure, the flow rate was only 0.4 mL/min. In contrast, the monolithic C18 column has much higher permeability and porosity, so the flow rate was 1 mL/min.

Nevertheless, for most analytes, we observed a lower w_0.05_ of the peaks using this C18 Polar column compared to the monolithic column, or the widths of the peaks were very similar on both columns (e.g., 2,3-dehydrosilychristin DHSCH, caffeic acid CAF or *p*-NP). The width of the peaks was very good for 2,3-dehydrosilybin DHSB, 2,3-dehydrosilybin-20-*O*-sulfate DHSB-S, and 2,3-dehydrosilybin-7,20-di-*O*-sulfate DHSB-SS, further for DHSCH, silychristin SCH, silybin SB, caffeic acid CAF, 4-methylcatechol MeCAT, and *p*-NP; good for 2,3-dehydrosilychristin-19-*O*-sulfate DHSCH-S, silychristin-19-*O*-sulfate SCH-S, protocatechuic acid PRO, 2,3,4-trihydroxybenzoic acid THB, catechol CAT, catechol-1-*O*-sulfate CAT-S, phloroglucinol PG, and its sulfate. Broader peaks were detected for five compounds, namely CAF-S, PRO-S, THB-S, MeCAT-S, and *p*-NP-S. In contrast, eight broad peaks were detected in the monolithic C18 column, most of which were sulfates (DHSB-S, DHSB-SS, SB-S, CAF-S, PRO-S, CAT-S, MeCAT-S, and PG-S). With the monolithic column, coelution of the peaks was observed in the case of *p*-NP-S and THB-S in the respective reaction mixtures. The retention times of the eluted compounds were up to 7 min. With the Polar C18 column, coelution of the peaks was observed only in the case of *p*-NP-S and MeCAT-S. The retention times of the eluted compounds were up to 9 min. Thus, when we compare the two C18 columns, the separation on the C18 Polar column shows narrower peaks and also a lower consumption of the mobile phase due to the lower flow rate (0.4 mL/min). In addition, this method is directly applicable in conjunction with a mass detector. The method with the monolithic column (M6) would require further modification in case of connection with the mass detector (flow rate 1 mL/min). The comparison of HPLC chromatograms of individual compounds is in Supplementary Material (Figures S13–S51).

### 2.4. Separation with Buffer in the Mobile Phase

As can be seen in the example of the separations of polyphenol sulfates on the PFP column and the C18 columns mentioned above, it is clear that in some cases, the mere acidification of the mobile phase is not sufficient to deionize ionizable compounds (broad peaks). Fine-tuning of the separation of highly polar sulfated polyphenols can be achieved by changes in the mobile phase, e.g., by choice of buffer, and thus consistent control of pH. The most commonly used buffers for HPLC with UV detection are acetate and phosphate. Since phosphate buffer is not compatible with MS detection, we chose ammonium acetate buffer with a pH of 3.8, and the buffer strength was set at 10 mM. All methods tested with 10 mM buffer were also tested with 5 mM concentration on several samples, but the width of the peaks was broader than with 10 mM buffer in all cases (data not shown).

Separation of quercetin (QUE), ampelopsin (AMP), luteolin (LUT), myricetin (MYR), isoquercitrin (ISQ), rutin (RUT), taxifolin (TAX), and their respective sulfates on PFP column using 10 mM ammonium acetate buffer and methanol as mobile phases (Method M1) showed good separation of most compounds (0.300 < w_0.05_ < 0.500). Only in two cases (QSS and AMP) was this range slightly exceeded (0.642 and 0.519, respectively). Moreover, the retention time of most compounds was shorter than in Method M3 (Table 3). No coelution of peaks was observed in the reaction mixtures. When we compare the separation of these compounds on the same PFP column, it is clear that we obtained better or equal separation with the acetate buffer method (Method M1) than with the method without acetate buffer (Method M3) in all cases except for ampelopsin (w_0.05_ 0.519 versus 0.314, Table 4).

In addition, we tested the separation of other polyphenolic compounds and their sulfates on this column so that we can compare it with other stationary phases used in this work. The separation of CAF, CAF-S, PRO, PRO-S, THB, THB-S, CAT, CAT-S, MeCAT-S, and PG-S was very good. The separation of DHSB-S, DHSCH, SCH, SCH-S, SB-S, MeCAT, *p*-NP, and *p*-NP-S was good; only the peaks of DHSB-SS and DHSCH-S were slightly broader (w_0.05_ was 0.525 and 0.576, respectively). Retention times of analytes ranged from 2 to 23 min; more than half of them were less than 10 min. No coelution of peaks in reaction mixtures was observed. From our previous unpublished experiments, we know that this gradient in method M1 is too fast for many phenolic acids, benzoic acids, and their sulfates. Therefore, we tried to modify this method. We reduced the initial concentration of mobile phase B from 40 to 20% and the final concentration to 50% (instead of the original 72%), and the length of the gradient was maintained (Method M2). However, we did not observe any significant improvement in w_0.05_, only the retention times were longer compared with Method 1.

Separation of phenolic compounds and their sulfates on the monolithic C18 column was also performed in 10 mM ammonium acetate buffer as the mobile phase (Method M5). The separation of many compounds was very good or good using this column (DHSB and its sulfates, DHSCH, DHSCH-S, CAF, PRO, PRO-S, THB, CAT, MeCAT, PG, and *p*-NP). However, for several compounds (SCH, SCH-S, SB, SB-S, CAF-S, CAT-S, MeCAt-S), the width of the peaks could not be determined because of their unusual shape (double hunch, see Section 2.5). The peak of THB-S was not caught on the stationary phase and eluted with the dead volume. The retention times of all compounds were up to 7 min. Coelution of *p*-NP and SCH-S was observed in the reaction mixture.

The Zic-pHILIC column was designed by the manufacturer for difficult separations of polar hydrophilic compounds. It is a polymer-based column with densely bound zwitterionic functional groups with a charged equilibrium of 1:1 and the lowest pH stability of 2. The mobile phases were acetonitrile and 10 mM ammonium acetate, both acidified with formic acid (pH 3.8), and the compounds were separated by gradient elution (Method M4). Almost all substances analyzed showed broad peaks except for DHSB, DHSCH-S, CAT-S, MeCAT-S, PG, and *p*-NP. Retention times were not measured for THB-S and CAT-S because the sulfated substance is likely degraded to the parent substance on the column. Retention times ranged from 0.9 to 9.5 min. Coelution of *p*-NP and SB, *p*-NP-S, and CAT was observed in the reaction mixture. The comparison of HPLC chromatograms of individual compounds is in Supplementary Material (Figures S19–S51).

### 2.5. Separation of Regioisomers and Stereoisomers

NMR analyses have shown that in some cases, multiple regioisomers are formed in enzymatic sulfation reactions, as in the case of QUE-S, QUE-SS, TAX-S, CAF-S, MeCAT-S, PRO-S, and THB-S. Separation of these regioisomers was achieved on a monolithic C18 column (Method M5) in CAF-S (peaks at 4.326 min and 4.494 min, resolution 1.251) and in MeCAT-S (double hunch peak at 4.362 min, resolution 0.540). The separation of two THB-S regioisomers was observed in Method M1 (peaks at 2.827 min and 3.033 min, resolution 1.944). The separation of two sulfated regioisomers was previously published on a C18 UHPLC column with MS/MS detection (3-phenylpropionic acid 4′-*O*-sulfate (RT 4.92 min) and 3-phenylpropionic acid 3′-*O*-sulfate (RT = 5.22 min) or caffeic acid-4′-*O*-sulfate (RT = 3.97 min) and caffeic acid-3′-*O*-sulfate) [2]. Baseline separation of disulfate regioisomers of myricetin was reported on the PFP column (mobile phase 0.1% trifluoroacetic acid in water and methanol) [8].

Since silybin is a mixture of two diastereoisomers, silybin A and B (in a ratio of approximately 1:1), we observed the separation of these two diastereoisomers in methods M1, M5, and M6. Sulfated diastereoisomers of silybin were separated by Methods M1 and M5. The resolution of the peaks was 1.365 (M1), 0.2 (M5), and 0.02 (M6). In the case of Method M4, we observed even three peaks (partially separated), and all of them have the same absorption maximum (286 nm). In this case, it is probably peak cleavage due to the unsuitability of the stationary phase for the separation of polyphenolic substances, as can be seen from the large peak width of most of the compounds examined. Although silychristin is also a mixture of two diastereoisomers A and B, we observed their separation only on the PFP column (M1). The separation of sulfated silychristin diastereoisomers was observed with the PFP column and Method M1 (Table 3). The separation of diastereoisomers of silychristin or silybin at the reverse phase is described in many papers, e.g., [25,26,27], but the separation of sulfated diastereoisomers have not been described so far.

### 2.6. Selection of the Best Method

In this study, we compared four types of stationary phases, namely pentafluorophenyl, ZICpHILIC, monolithic C18, and C18 stationary phases, with treatment for better polar retention. We combined these stationary phases with either a mobile phase without a buffer or with a buffer. The combination of pentafluorophenyl stationary phase and 10 mM acetate buffer/methanol in gradient elution proved to be the best. This resulted in sharp peaks for almost all test compounds without tailing and very good separation of all components in the mixture for up to 20 min (Figure 5). The other columns/mobile phases tested were only suitable for some of the compounds analyzed. The retention times and peak widths of all compounds tested are shown in Table 3 and Table 4. The peak shapes and comparisons of HPLC chromatograms of individual compounds in all methods tested can be found in Supplementary Materials (Figures S13–S51).

### 2.7. Method Validation

Due to the large number of samples measured and columns tested, one representative from the group of sulfated phenols, phenolic acids, flavonoids, and flavonolignans, namely 4-methylcatechol sulfate (MeCAT-S), caffeic acid sulfate (CAF-S), ampelopsin sulfate (AMP-S), and silychristin sulfate (SCH-S), was selected for the validation of Method M1 (the most universal PFP column and mobile phase for all samples tested). The linearity, limit of detection, the limit of quantification, precision, accuracy, recovery and repeatability are summarized in Table 5.

The calibration curves were linear in the range from 0.625 to 50 mM for all analytes. The correlation coefficients were greater than 0.9997 in all cases (except AMP-S, 0.9977), demonstrating a high degree of correlation and good linearity of the method. LOD and LOQ ranged from 0.032 to 1.680 mM. This indicates that our method has adequate sensitivity. The ranges of %RSD parameters for repeatability (intra-day precision) and intermediate (inter-day) precision were 1.55 to 3.63 and 2.18 to 9.04, respectively. The accuracy ranges were from 1.3 (MeCAT-S) to 3.3% (SCH-S), and recoveries were in the range of 103–104% for all tested samples. Sample stability was evaluated by storing unprocessed samples at ambient temperature up to 24 h and freeze/thaw cycles after three cycles at −18 °C. The experiments indicated that all four analytes tested were stable in the period of 24 h, as the recoveries ranged between 97–102% for SCH-S, 94–104% for CAF-S, 98–103% for AMP-S, and 97–102% for MeCAT-S.

## 3. Materials and Methods

### 3.1. Material

Acetonitrile, methanol, formic acid (all VWR chemicals, Stříbrná Skalice, Czech Republic analytical grade), deionized water (Ultrapure, Watrex, Prague, Czech Republic), ammonium acetate (Lach-Ner, Neratovice, Czech Republic), ampelopsin (Herb Nutritionals, Shanghai, China), 3,4-dihydroxycinamic acid (caffeic acid), *p*-nitrophenol sulfate, 2,3,4-trihydroxybenzoic acid, 3,4-dihydroxybenzoic (protocatechuic) acid, catechol (all Acros Organics, Thermo Fisher Scientific, Waltham, MA, USA), 4-methylcatechol (Aldrich, Merck KGaA, Darmstadt, Germany), quercetin (Sigma, Merck KGaA, Darmstadt, Germany), phloroglucinol (Alfa Aesar, Haverhill, MA, USA), *p*-nitrophenol, luteolin, and myricetin (abcr GmbH, Karlsruhe, Germany). Standards of silybin, 2,3-dehydrosilybin, and silychristin were prepared and fully characterized in the Laboratory of Biotransformation, Institute of Microbiology, Prague, Czech Republic [28,29,30].

The description of the preparation and full characterization (NMR, HRMS, and HPLC) of the following sulfates have already been published: silybin A-20-*O*-sulfate and silybin B-20-*O*-sulfate (SB-S, 50:50) [10], silychristin-19-*O*-sulfate (SCH-S), 2,3-dehydrosilybin-20-*O*-sulfate (DHSB-S), 2,3-dehydrosilychristin-19-*O*-sulfate (DHSCH-S) [13], quercetin-3′-*O*-sulfate and quercetin-4′-*O*-sulfate (QUE-S, 75:25) [11,12,19], rutin-4′-*O*-sulfate (RUT-S), taxifolin-4′-*O*-sulfate and taxifolin-3′-*O*-sulfate (TAX-S, 80:20), isoquercitrin-4′-*O*-sulfate (ISQ-S) [11,19], ampelopsin-4′-*O*-sulfate (AMP-S), luteolin-3′-*O*-sulfate (LUT-S), luteolin-7, 3′- and 7, 4′-di-*O*-sulfates (LUT-SS), myricetin-4′-O-sulfate (MYR-S), myricetin-di-7, and 4′-*O*-sulfate (MYR-SS) [8].

### 3.2. Preparation of the Enzyme

The aryl sulfotransferase from *Desulfitobacterium hafniense* used for the sulfation was prepared as described previously in our works [11,12].

### 3.3. General Method for the Preparation of Sulfates

The substrate (MeCAT, PRO, THB, CAF, CAT, PG; 200 mg of each, 1 eq) was dissolved in 5 mL of acetone in a flask and then *p*-NPS (25 mg/mL, 1 or 2 eq in 100 mM Tris-glycine buffer pH 8.9), 24 mL of Tris-glycine buffer, and 2 mL of AST enzyme (360 U/mL) were added. The reaction mixture was then incubated under an inert atmosphere (Ar) at 30 °C for 5 h. The monitoring of the reaction was performed using TLC (mobile phase ethyl acetate/chloroform/trifluoroacetic acid, 16/1/0.01). After incubation, the reaction mixture was heated up to 95 °C to terminate the enzymatic reaction and stored at −20 °C until purification.

### 3.4. Purification of Sulfates

In the case of MeCAT, PRO, THB, CAF, and CAT, the reaction mixture was partially evaporated in a rotary evaporator to remove acetone from the mixture. The pH was adjusted to 7.5–7.7 using formic acid, and then the mixture was extracted with ethyl acetate (3 × 50 mL) to remove *p*-nitrophenol (control by TLC, mobile phase ethyl acetate/chloroform/trifluoroacetic acid, 16/1/0.01). The aqueous phase was then evaporated, dissolved in 2–5 mL of 80% methanol, centrifuged (5000 × rpm, 20 min), and loaded onto a Sephadex LH-20 column (GE Healthcare Bio-Sciences, Uppsala, Sweden; 30 g of dry weight, 3 cm i.d.) with 80% methanol as a mobile phase (0.2 mL/min). The elution time was usually 2 days, and the fraction detection was performed by TLC (ethyl acetate/chloroform/trifluoroacetic acid, 16/1/0.01). Purification of the phloroglucinol reaction mixture was performed using preparative HPLC (Section 3.5) using an ASAHIPAK GS-310 20F column (Shodex, Munich, Germany). The reaction mixture (100 mg) was dissolved in 1 mL of 50% methanol, filtered, and injected onto the column (5 mL/min, 25 °C, detection at 254 and 369 nm). The fractions containing the desired product were then joined, fully evaporated, and lyophilized. Low purity fractions were re-purified using the same methodology.

### 3.5. Preparative HPLC

The preparative HPLC (Shimadzu, Kyoto, Japan) system consisted of an LC-8A high-pressure pump with an SPD-20A dual-wavelength detector (with preparative cell), FRC-10A, and fraction collector. The system was connected to a PC using a CBM20A command module and controlled by the LabSolution 1.24 SPI software suite supplied with the instrument.

### 3.6. Mass Spectrometry (MS)

The samples were dissolved in MeOH and introduced into the mobile phase flow (MeOH/H2O 4:1; 100 µL/min) using a 2 µL loop. Spray voltage, capillary voltage, tube lens voltage, and capillary temperature were 4.0 kV, −16 V, −120 V, and 275 °C, respectively.

### 3.7. Nuclear Magnetic Resonance (NMR)

NMR spectra were recorded on a Bruker Avance III 600 MHz and 400 MHz spectrometers at 30 °C in dimethylsulfoxide (DMSO-*d*_6_); residual solvent signal (*δ*_H_ 2.499 ppm, *δ*_C_ 39.46 ppm) served as an internal standard. NMR experiments: ^1^H NMR, ^13^C NMR, gCOSY, gHSQC, and gHMBC were performed using the standard manufacturer’s software. The position of sulfate attachment was determined using typical changes in chemical shifts of the attached and adjacent carbons (compared with starting acceptors) as described in [11].

### 3.8. Analytical HPLC System

The Shimadzu Prominence LC analytical system comprised Shimadzu CBM-20A system controller, Shimadzu LC-20AD binary HPLC pump, Shimadzu CTO-10AS column oven, Shimadzu SIL-20ACHT cooling autosampler, and Shimadzu SPD-20MA diode array detector (Shimadzu, Kyoto, Japan).

### 3.9. Analytical Columns and Mobile Phases

Kinetex PFP column (150 × 4.6 mm, 5 µm), guard column PFP (4 × 3 mm, 5 µm), both Phenomenex (USA); Method M1: mobile phase A = 10 mM ammonium acetate, 0.1% HCOOH, pH 3.8; mobile phase B = 100% MeOH; gradient elution: 0 min 40% B, 0–20 min 40–72% B, 20–21 min 72–40% B, 21–24 min 40% for equilibration of the column; flow rate 0.6 mL/min, 45 °C, PDA detection (200–400 nm), the wavelength of the absorption maximum of the respective compound was extracted. The method with the same mobile phases, flow rate, temperature, and detection but a different gradient was used for the analysis of phenolic acids PRO, THB, CAF, and also for CAT, MeCAT, PG, respectively. (Method M2): 0 min 20% B, 0–20 min 20–50% B, 20–21 min 50–20% B, 21–24 min 20% B for equilibration of the column. Another method (Method M3) that has also been tested with this column: mobile phase A = 0.1% TFA (pH = 2.0), B = 100% MeOH; gradient elution 0 min 40% B, 0–25 min 40–80% B, 25–26 min 80–40% B, 26–28 min 40% for equilibration of the column; flow rate 0.6 mL/min, 45 °C.

ZicpHILIC column (100 × 2.1 mm, 5 µm), guard column ZicHILIC (20 × 2.1 mm, 5 µm), both Merck (DE); Method M4: mobile phase A = 100% acetonitrile, 0.1% HCOOH; mobile phase B = 10 mM ammonium acetate, 0.1% HCOOH, pH 3.8; gradient elution: 0 min 5% B, 0–7.5 min 5–20% B, 7.5–10 min 20% B, 10–12 min 20–5% B, 12–15 min 5% B, 15–17 5% B for equilibration of the column; flow rate 0.4 mL/min, 25 °C, PDA detection (200–400 nm), the wavelength of the absorption maximum of the respective compound was extracted.

Chromolith RP 18e column (100 × 3 mm, monolith), guard column Chromolith RP 18-e (5 × 4.6 mm, monolitith), both Merck (DE); Method M5: mobile phase A = 10 mM ammonium acetate, 0.1% HCOOH, pH 3.8, mobile phase B = 100% MeOH; gradient elution: 0–2 min 5% B, 2–7 min 5–90% B, 7–8 min 90%B, 8–11 min 90–5% B, 11–14 min 5% B for equilibration of the column; flow rate 1 mL/min, 25 °C, PDA detection (200–400 nm), the wavelength of the absorption maximum of the respective compound was extracted. Another method (Method M6) has also been tested with this column: mobile phase A = 5% acetonitrile, 0.1% HCOOH, B = 80% acetonitrile, 0.1% HCOOH; gradient elution 0–5 min 0–30% B, 5–7 min 30–0% B, 7–9 min 0% B for equilibration of the column; flow rate 1.0 mL/min, 25 °C.

Luna Omega Polar C18 column (100 × 2.1 mm, 3 µm), guard column Polar C18 for 2.1 mm ID, both Phenomenex (USA); Method M7: mobile phase A = 5% acetonitrile, 0.1% HCOOH, mobile phase B = 80% acetonitrile, 0.1% HCOOH; gradient elution: 0–7 min 0–90% B, 7–8 min 90% B, 8–11 min 90–0% B, 11–14 min 0% B for equilibration of the column; flow rate 0.4 mL/min, 45 °C, PDA detection (200–400 nm), the wavelength of the absorption maximum of the respective compound was extracted.

All of the above methods, where 10 mM ammonium acetate was used in the mobile phase, were also tested with 5 mM ammonium acetate under the same conditions. An overview of the methods used is in Table 2.

### 3.10. Sample Preparation

Samples of individual sulfates were dissolved in water or parent compounds in MeOH/water (1/1, *v/v*) (1 mg/mL), centrifugated, and injected (1 µL). The reaction mixtures (10 µL) were diluted by water (50 µL), centrifuged, and injected (1 µL).

### 3.11. Method Validation

Four representatives from the group of phenol, phenolic acid, flavonoid, and flavonolignan sulfates, namely 4-methylcatechol sulfate (MeCAT-S), caffeic acid sulfate (CAF-S), ampelopsin sulfate (AMP-S), and silychristin sulfate (SCH-S), were selected for validation on the PFP column (the most universal column for all samples tested, Method M1, see Table 2).

#### 3.11.1. Linearity

Linearity was evaluated by measuring five concentrations of each analyte in duplicates. The concentrations of the analytes prepared in the volumetric flasks were 50, 25, 12.5, 6.25, and 0.625 mM. The results were examined for a linear relationship by plotting the peak area and the corresponding concentrations of the analyte, followed by linear least squares regression and calculation of the slope and correlation coefficient.

#### 3.11.2. Limit of Detection (LOD) and Limit of Quantification (LOQ)

The values of LOQ and LOD were determined according to the following equations: LOD = 3 × noise/slope of the corresponding calibration curve, LOQ = 10 × noise/slope of the corresponding calibration curve.

#### 3.11.3. Precision and Repeatability

Intra-day precision (repeatability) and inter-day precision (intermediate precision) were calculated by analyzing four selected samples at the concentration of 12.5 mM. Results were expressed as the relative standard derivation (RSD). Repeatability measurement was performed on the same day in six replicates; the intermediate precision was measured on six different days.

## 4. Conclusions

We have developed a robust HPLC analytical method suitable for the separation of enzymatic sulfation reaction mixtures of flavonoids, dehydroflavonoids, phenolic acids, and catechols with PDA detection. This method is based on the combination of pentafluorophenyl stationary phase and 10 mM acetate buffer/methanol in gradient elution. Moreover, the low flow rate (0.6 mL/min) and the absence of phosphate buffer and/or ion-pairing reagents in the mobile phase make the method directly applicable in combination with mass detection. Last but not least, four authentic standards of 2,3,4-trihydroxybenzoic acid sulfates, catechol sulfate, 4-methylcatechol sulfate, and phloroglucinol sulfate were prepared in this work.

## Figures and Tables

**Figure 5:**
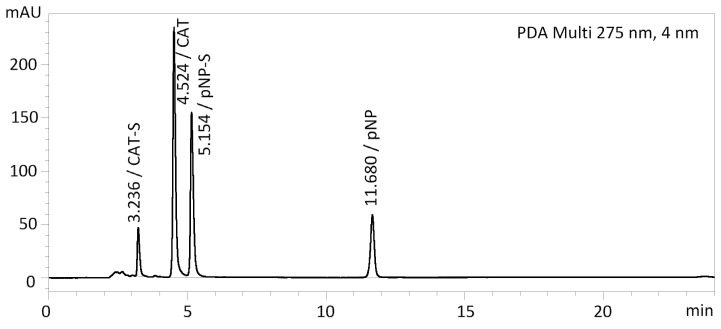
HPLC chromatogram—an example of typical composition of the enzymatic reaction mixture to be analyzed: *p*-NP, *p*-NP-S, parent compound (CAT), and its sulfate (CAT-S), Method M1.

**Table 1:** An overview of HPLC methods for sulfate separation published to date and their parameters.

Column	C18									C18 ^a^	HILIC ^b^		Phenyl ^a^	PFP ^c^			Polyamine
Phosphate in mobile phase	+	+	+	-	-	-	-	+	+	-	-	-	-	-	-	-	+
Ion-pairing reagent	-	+	+	-	-	-	-	+	+	-	-	-	-	-	-	-	-
MS/MS, QTof detection	-	-	-	-	-	+	-	+	-	+	+	-	+	-	-	-	Fluorescence
Long separation time (≥ 20 min)	? ^d^	+	+	+	+	-	-	+	-	+	+	-	-	+	+	-	-
Tailing peaks, coelution	?	-	+	?	-	?	+	-	+	-	+	+	+	+	+	+	+
Reference	[16]	[6]	[7]	[24]	[23]	[20]	[11]	[15]	[9]	[2]	[18]	[22]	[19]	[21]	[8]	[12,13]	[17]

^a^ Ultra-high performance liquid chromatography (UHPLC), ^b^ hydrophilic interaction chromatography, ^c^ pentafluorophenyl, ^d^ information not provided in the reference.

**Table 2:** An overview of the columns used and separation conditions.

Stationary Phase	Method Number	Mobile Phase A	Mobile Phase B	Flow Rate [mL/min]	T [°C]	Gradient
PFP	M1	10 mM CH_3_COONH_4_/HCOOH (100/0.1, *v*/*v*)	MeOH	0.6	45	0 min 40% B, 0–20 min 40–72% B, 20–21 min 72–40% B, 21–24 min 40%
	M2	10 mM CH_3_COONH_4_/HCOOH (100/0.1, *v*/*v*)	MeOH	0.6	45	0 min 40% B, 0–20 min 20–50% B, 20–21 min 50–20% B, 21–24 min 20%
	M3	H_2_O/CH_3_COOF_3_ (100/0.1, *v*/*v*)	MeOH	0.6	45	0 min 40% B, 0–25 min 40–80% B, 25–26 min 80–40% B, 26–28 min 40%
ZICpHILIC	M4	AcCN/HCOOH (100/0.1, *v*/*v*)	10 mM CH_3_COONH_4_/HCOOH (100/0.1, *v*/*v*)	0.4	25	0 min 5% B, 0–7.5 min 5–20% B, 7.5–10 min 20% B, 10–12 min 20–5% B, 12–15 min 5% B, 15–17 5% B
C18	M5	10 mM CH_3_COONH_4_/HCOOH (100/0.1, *v*/*v*)	MeOH	1	25	0–2 min 5% B, 2–7 min 5–90% B, 7–8 min 90%B, 8–11 min 90–5% B, 11–14 min 5% B
	M6	AcCN/H_2_O/HCOOH(5/95/0.1, *v/v* )	AcCN/H_2_O/HCOOH (80/20/0.1)	1	25	0–5 min 0–30% B, 5–7 min 30–0% B, 7–9 min 0% B
C18 Polar	M7	H_2_O, HCOOH (100/0.1, *v*/*v*)	AcCN/H_2_O/HCOOH (80/20/0.1)	0.4	25	0–7 min 0–90% B, 7–8 min 90% B, 8–11 min 90–0% B, 11–14 min 0% B

**Table 3:** Comparison of retention times and peak widths of selected analytes using different columns and methods.

Stationary Phase	PFP ^a^				ZICpHILIC ^b^		C18				C18-Polar	
Method ^c^	M1		M2		M4		M5		M6		M7	
Analyte ^d^	t_R_^e^	w_0.05_^f^	t_R_^e^	w_0.05_^f^	t_R_^e^	w_0.05_^f^	t_R_^e^	w_0.05_^f^	t_R_^e^	w_0.05_^f^	t_R_^e^	w_0.05_^f^
	[min]											
DHSB	23.101	0.329	-	-	1.160	0.251	7.293	0.146	7.039	0.177	9.032	0.143
DHSB-S	17.385	0.347	-	-	3.636	0.849	6.914	0.189	6.771	0.608	7.904	0.234
DHSB-SS	8.280	0.525	-	-	8.853	0.588	6.097	0.392	3.180	1.701	8.490	0.146
DHSCH	17.010	0.425	-	-	2.025	1.209	6.691	0.108	6.957	0.101	7.573	0.125
DHSCH-S	12.060	0.576	-	-	5.448	0.465	6.258	0.177	6.771	0.478	6.768	0.322
SCH	12.449, 13.285 ^i^	0.299, 0.312	-	-	3.047	1.210	6.002	n.d. ^g^	5.753	0.156	7.413	0.132
SCH-S	6.176, 6.654 ^h^	0.338	-	-	6.332	0.540	5.552	n.d. ^g^	4.985	0.393	6.313	0.395
SB	15.202, 15.521 ^i^	n.d. ^g^	-	-	1.094	0.647 ^i^	6.480, 6.514 ^i^	n.d. ^g^	4.141, 4.225 ^i^	n.d. ^g^	7.410	0.186
SB-S	13.728, 14.479 ^h^	0.287 0.415	-	-	2.470, 3.007, 4.365 ^j^	n.d. ^g^	6.487, 6.579 ^h^	n.d. ^g^	3.165	0.981	6.555	0.186
CAF	5.150	0.238	12.527	0.356	2.540	0.660	4.954	0.102	3.087	0.211	5.987	0.270
CAF-S	3.720	0.267	9.233	0.671	4.194	0.528	4.326, 4.494 ^j^	n.d. ^g^	5.847	2.555	5.366	1.040
PRO	3.721	0.270	7.213	0.337	3.184	0.787	2.816	0.410	1.822	0.237	3.653	0.471
PRO-S	2.990	0.225	5.541	0.403	6.255	0.558	2.104	0.276	4.697	2.579	2.500	0.920
THB	3.568	0.246	6.240	0.368	4.456	0.638	1.944	0.175	2.058	0.259	4.122	0.383
THB-S	2.693, 3.033 ^j^	0.329, 0.266	4.949	0.317	n.d. ^k^	-	n.d. ^l^	-	0.718	0.050	2.966	0.582
CAT	4.578	0.256	7.453	0.350	1.257	0.270	2.920	0.356	2.317	0.246	4.331	0.403
CAT-S	3.240	0.207	4.938	0.386	n.d. ^k^		4.290	n.d. ^g^	5.109	2.564	7.627	0.305
MeCAT	5.978	0.335	11.944	0.508	0.963	n.d. ^g^	4.946	0.226	3.769	0.300	6.302	0.261
MeCAT-S	4.112	0.249	7.910	0.524	1.049	0.314	4.362 ^j^	n.d. ^g^	6.578	2.546	5.536	0.507
PG	2.906	0.205	4.004	0.235	4.641	0.430	1.180	0.288	1.039	0.197	1.641	0.310
PG-S	2.503	0.177	3.339	0.266	9.500	0.732	1.465	0.567	2.683	1.534	1.265	0.308
*p*NP	11.528	0.338	20.542	0.385	0.984	0.273	5.609	0.169	5.031	0.221	7.274	0.278
*p*NP-S	4.930	0.457	10.401	0.498	1.494	0.600	5.216	0.900	0.796	0.140	5.567	0.621

^a^ Kinetex pentafluorophenyl, ^b^ hydrophilic interaction chromatography, ^c^ for details on the individual methods, see Table 2, ^d^ full names and structures of the analytes are shown at Figure 1, Figure 2 and Figure 3, ^e^ retention time, ^f^ the width of the peak in 5% of its height, ^g^ the peak shape did not allow the determination of w_0.05_, ^h^ separation of sulfated stereoisomers A and B, ^i^ partial separation of stereoisomers A and B, ^j^ partial separation of sulfated regioisomers, ^k^ the compound was decomposed during the analysis, only the parent compound without sulfate was detected, ^l^ the compound was not caught on the column and eluted with a dead volume. Dark green means w_0.05_ < 0.300, light green means 0.300 < w_0.05_ < 0.500, and red means w_0.05_ > 0.500.

**Table 4:** Comparison of retention times and peak widths of selected analytes using PFP column with (M1) and without buffer (M3).

Method	M1 ^a^		M3 ^b^	
Analyte ^c^	t_R_^d^ [min]	w_0.05_^e^ [min]	t_R_^d^ [min]	w_0.05_^e^ [min]
QUE	16.127	0.385	17.470	0.554
QUE-S	12.139	0.397	15.084	0.632
QUE-SS	6.014	0.642	11.084	1.703
AMP	5.480	0.519	6.062	0.314
AMP-S	6.007	0.352	7.449	0.437
LUT	17.916	0.427	20.010	0.422
LUT-S	13.340	0.377	16.741	0.501
LUT-SS	12.830	0.362	13.595	1.426
MYR	12.716	0.390	14.014	0.410
M-S	8.566	0.478	15.877	0.542
M-SS	4.602	0.400	12.497	1.398
ISQ	9.397	0.344	9.615	0.312
ISQ-S	6.681	0.311	8.247	0.381
RUT	8.869	0.342	9.156	0.322
RUT-S	6.055	0.261	7.175	0.287
TAX	7.380	0.285	7.767	0.466
TAX-S	5.582	0.321	7.557	0.368
*p*NP	11.528	0.338	13.414	0.367
*p*NP-S	4.930	0.457	6.751	0.295

^a^ With ammonium acetate buffer, ^b^ with 0.1% TFA (for details, see Table 2), ^c^ full names and structures of the analytes are shown in Figure 1, Figure 2 and Figure 3, ^d^ retention times, ^e^ width of the peak in 5% of its height; dark green means w_0.05_ < 0.300, light green means 0.300 < w_0.05_ < 0.500, and red means w_0.05_ > 0.500.

**Table 5:** The linearity, limit of detection (LOD), limit of quantification (LOQ), intermediate precision, repeatability, accuracy, and recovery for four representatives of sulfated phenol (MeCAT-S), phenolic acid (CAF-S), flavonoids (AMP-S), and flavonolignans (SCH-S).

Sample	Regression Equation	R^2^	LOD [mM]	LOQ [mM]	Repeatability [%]^a^	Intermediate Precision [%] ^a^	Accuracy [%] ^b^	Recovery [%]
MeCAT-S	y = 161,221 × c + 17,188	0.9999	0.032	0.108	1.55	2.18	1.3	104
CAF-S	y = 26,266 × c + 4120	0.9998	0.560	1.680	3.63	8.13	2.2	104
AMP-S	y = 96,081 × c	0.9977	0.061	0.202	1.74	6.30	2.4	103
SCH-S	y = 127,596 × c + 27,799	0.9999	0.340	1.020	1.78	9.04	3.3	105

R^2^ correlation coefficient; ^a^ expressed as relative standard deviation, n = 6; ^b^ expressed as relative standard deviation, n = 3.

## Data Availability

The data presented in this study are available in the article or supplementary material.

## Supplementary Materials

The following are available online at https://www.mdpi.com/article/10.3390/ijms23105743/s1.

## Abbreviations

AMP	ampelopsin
AMP-S	ampelopsin-4′-*O*-sulfate
CAF	caffeic acid
CAF-S	caffeic acid 3- and 4-*O*-sulfate (69:31)
CAT	catechol
CAT-S	catechol-*O*-sulfate
DHSB	2,3-dehydrosilybin
DHSB-S	2,3-dehydrosilybin-20-*O*-sulfate
DHSB-SS	2,3-dehydrosilybin-7,20-di-*O*-sulfate
DHSCH	2,3-dehydrosilychristin
DHSCH-S	2,3-dehydrosilychristin-19-*O*-sulfate
ISQ	isoquercitrin
ISQ-S	isoquercitrin-4′-*O*-sulfate
LUT	luteolin
LUT-S	luteolin-3′-*O*-sulfate
LUT-SS	luteolin-7,3′- and 7, 4′-di-*O*-sulfates (82:12)
MeCAT	4-methylcatechol
MeCAT-S	4-methylcatechol-1- and 2-*O*-sulfate (64:36)
MYR	myricetin
MYR-S	myricetin-4′-*O*-sulfate
MYR-SS	myricetin-7,4′-di-*O*-sulfate
*p*-NP	*p*-nitrophenol
*p*-NPS	*p*-nitrophenyl sulfate
PG	phloroglucinol
PG-S	phloroglucinol-*O*-sulfate
PRO	protocatechuic acid
PRO-S	protocatechuic acid 3- and 4-*O*-sulfates (70:30)
QUE	quercetin
QUE-S	quercetin-3′- and 4′-*O*-sulfate (75:25)
QUE-SS	quercetin- 3′,4′-, 7,3′- and 7, 4′-di-*O*-sulfate
RUT	rutin
RUT-S	rutin-4′-*O*-sulfate
SB	silybin A and B (50:50)
SB-S	silybin A-20-*O*-sulfate and silybin B-20-*O*-sulfate (50:50)
SCH	silychristin A and B (90:10)
SCH-S	silychristin-19-*O*-sulfate
THB	2,3,4-trihydroxybenzoic acid
TX	taxifolin
TX-S	taxifolin-4′- and 3′-*O*-sulfate (80:20)
