# Supplementary material for: Sulfated Phenolic Substances: Preparation and Optimized HPLC Analysis
Source: Int J Mol Sci. 2022 May 20;23(10):5743. doi: 10.3390/ijms23105743 (PMC9147169; doi:10.3390/ijms23105743)
Supplement: Supplementary file 1 [file ijms-23-05743-s001.zip › ijms-1731237-supplementary.pdf]

International Journal of *Molecular Sciences*

MDPI

Supplementary Materials

Sulfated Phenolic Substances: Preparation and Optimized HPLC Analysis

Lucie Petrásková[1], Kristýna Káňová[1,2], Katerina Brodsky[1,2], Anastasiia Hetman[1,3], Barbora Petránková[1,4], Helena Pelantová[1], Vladimír Křen[1], Kateřina Valentová[1,*]

TABLE OF CONTENTS

1 ABBREVIATIONS OF THE COMPOUNDS USED IN THE STUDY 2

2 STRUCTURAL CHARACTERIZATION OF PREPARED COMPOUNDS 3

2.1 4-Methylcatechol-*O*-sulfates (MeCAT-S) 3

2.2 Protocatechuic acid *O*-sulfates (PRO-S) 4

2.3 2,3,4-Trihydroxybenzoic acid *O*-sulfates (THB-S) 6

2.4 Caffeic acid *O*-sulfates (CAF-S) 8

2.5 Catechol-1-*O*-sulfate (CAT-S) 9

2.6 Phloroglucinol-*O*-sulfate (PG-S).. 10

3 RETENTION TIMES, WIDTHS OF THE PEAKS AND ABSORPTION MAXIMA OF THE COMPOUNDS 12

3.1 Methods without buffer in the mobile phase 12

3.2 Methods with ammonium acetate buffer in the mobile phase 15

4 COMPARISON OF HPLC CHROMATOGRAMS FOR INDIVIDUAL COMPOUNDS .. 19

International Journal of
Molecular Sciences

1 ABBREVIATIONS OF THE COMPOUNDS USED IN THE STUDY

AMP	ampelopsin
AMP-S	ampelopsin-4′-O-sulfate
CAF	caffeic acid
CAF-S	caffeic acid 3- and 4-*O*-sulfate (69:31)
CAT	catechol
CAT-S	catechol-*O*-sulfate
DHSB	2,3-dehydrosilybin
DHSB-S	2,3-dehydrosilybin-20-*O*-sulfate
DHSB-SS	2,3-dehydrosilybin-7,20-di-*O*-sulfate
DHSCH	2,3-dehydrosilychristin
DHSCH-S	2,3-dehydrosilychristin-19-*O*-sulfate
ISQ	isoquercitrin
ISQ-S	isoquercitrin-4′-O-sulfate
LUT	luteolin
LUT-S	luteolin-3′-*O*-sulfate
LUT-SS	luteolin-7,3′- and 7, 4′-di-*O*-sulfates (82:12)
MeCAT	4-methylcatechol
MeCAT-S	4-methylcatechol-1- and 2-O-sulfate (64:36)
MYR	myricetin
MYR-S	myriceti n-4′-*O*-sulfate
MYR-SS	myricetin-7,4′-di-O-sulfate
p-NP	*p*-nitrophenol
p-NPS	*p*-nitrophenyl sulfate
PG	phloroglucinol
PG-S	phloroglucinol-*O*-sulfate
PRO	protocatechuic acid
PRO-S	protocatechuic acid 3- and 4-*O*-sulfates (70:30)
QUE	quercetin
QUE-S	quercetin-3′- and 4′-*O*-sulfate (75:25)
QUE-SS	quercetin- 3′,4′-, 7,3′- and 7, 4′-di-*O*-sulfate
RUT	rutin
RUT-S	rutin-4′-*O*-sulfate
SB	silybin A and B (50:50)
SB-S	silybin A-20-*O*-sulfate and silybin B-20-*O*-sulfate (50:50)
SCH	silychristin A and B (90:10)
SCH-S	silychristin-19-*O*-sulfate
THB	2,3,4-trihydroxybenzoic acid
THB	2,3,4-trihydroxybenzoic acid
TX	taxifolin
TX-S	taxifolin-4′- and 3′-*O*-sulfate (80:20)

2 STRUCTURAL CHARACTERIZATION OF PREPARED COMPOUNDS

NMR spectra were recorded on a Bruker Avance III 600 MHz and 400 MHz spectrometer at 30 °C in dimethylsulfoxide (DMSO-d_6); residual solvent signal (δ_H 2.499 ppm, δ_C 39.46 ppm) served as an internal standard. NMR experiments: ^{1}H NMR, ^{13}C NMR, gCOSY, gHSQC, and gHMBC were performed using the standard manufacturer's software.
The position of sulfate attachment was determined using typical changes in chemical shifts of the attached and adjacent carbons (compared with starting acceptors) as described in [1].

2.1 4-Methylcatechol-O-sulfates (MeCAT-S)

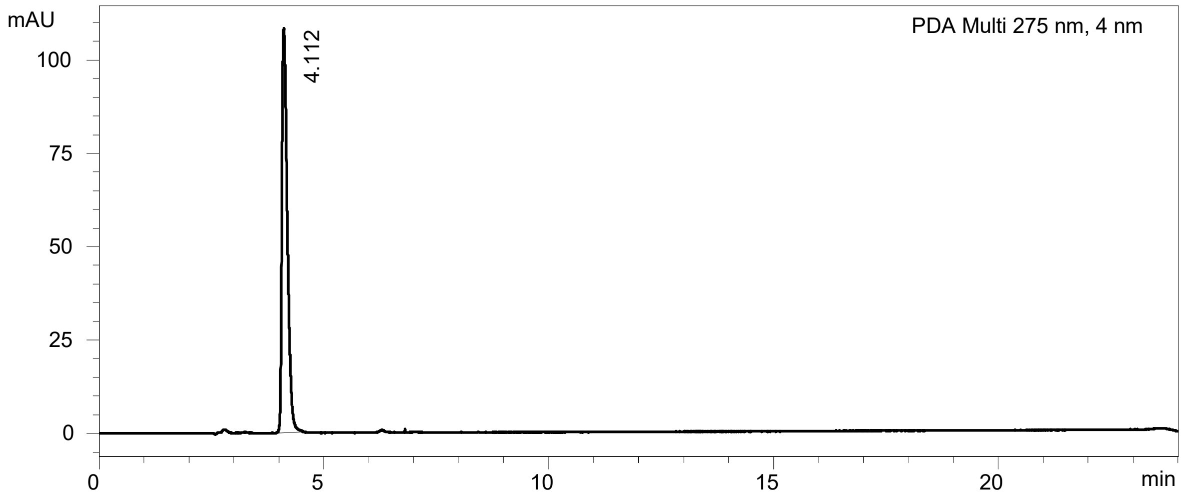

Figure S1. HPLC chromatogram of 4-methylcatechol-*O*-sulfates (MeCAT, regioisomers), method M1 (RT= 4.112 min, 99% purity)

Table S8a. ^{13}C and ^{1}H NMR data of 4-methylcatechol-1-*O*-sulfate (600.23 MHz for ^{1}H, 150.93 MHz for ^{13}C, DMSO-d_6, 30 °C)

Atom	δ_C [ppm]	*m*	δ_H [ppm]	*n*(H)	*m*	*J* [HZ]
1	138.58	S	-	0	-	-
2	148.82	S	-	0	-	-
3	117.70	D	6.630	1	dq	2.1, 0.8
4	134.11	S	-	0	-	-
5	119.78	D	6.538	1	ddq	8.1, 2.1, 0.8
6	122.88	D	6.937	1	d	8.1
1-Me	20.36	Q	2.188	3	m	-

δ - chemical shift, *m* – multiplicity, *n* – number of hydrogens, *J* – interaction constant

International Journal of
Molecular Sciences

MDPI

Table S8b. ^{13}C and ^{1}H NMR data of 4-methylcatechol-2-*O*-sulfate (600.23 MHz for ^{1}H, 150.93 MHz for ^{13}C, DMSO-d_6, 30 °C)

Atom	δ_C [ppm]	*m*	δ_H [ppm]	*n*(H)	*m*	*J* [HZ]
1	146.76	S	-	0	-	-
2	140.46	S	-	0	-	-
3	123.54	D	6.904	1	dq	2.1, 0.8
4	128.06	S	-	0	-	-
5	125.18	D	6.749	1	ddq	8.1, 2.1, 0.8
6	116.88	D	6.693	1	d	8.1
1-Me	19.95	Q	2.175	3	m	-

δ - chemical shift, *m* – multiplicity, *n* – number of hydrogens, *J* – interaction constant

molar ratio 1-*O*-sulfate : 2-*O*-sulfate = 64 : 36

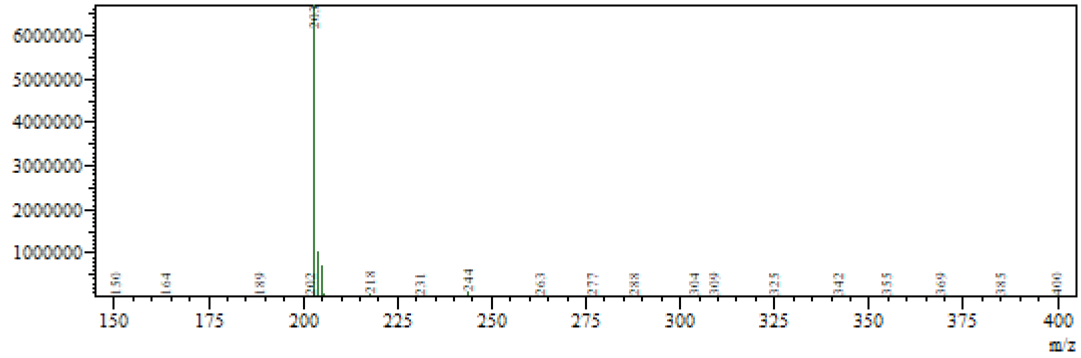

Figure S2. MS-ESI$^-$ spectrum of 4-methyl-catechol-*O*-sulfate ($[M - H]^-$, *m/z* calcd for $C_7H_7O_5S$: 203.01; found: 203)

2.2 *Protocatechuic acid* O-*sulfates (PRO-S)*

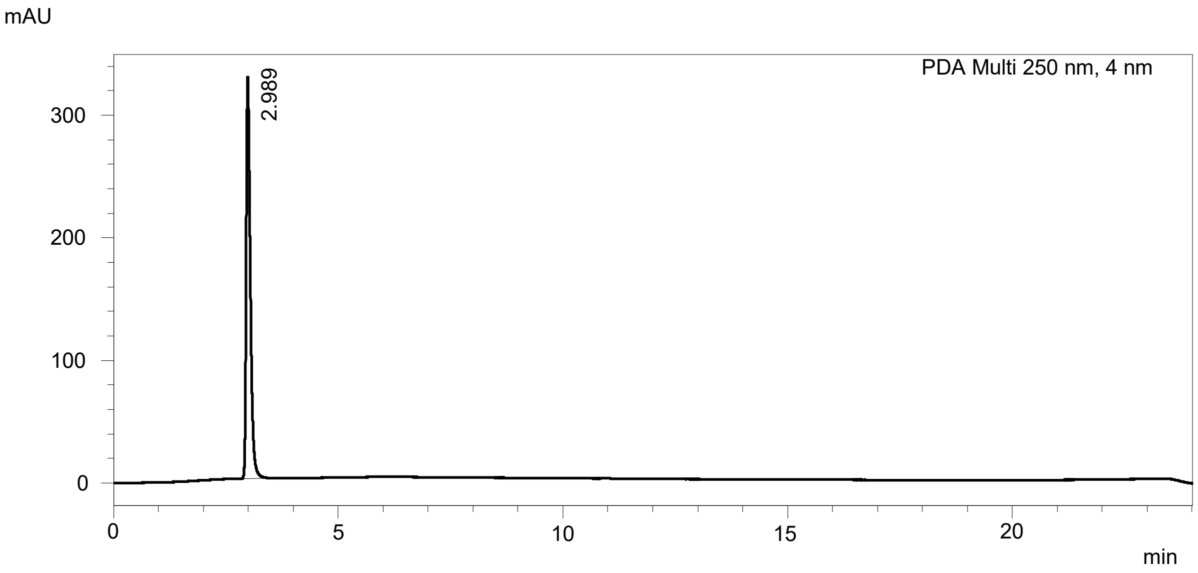

Figure S3. HPLC chromatogram of protocatechuic acid *O*-sulfates (PRO-S, regioisomers), method M1 (RT = 2.989 min, 99% purity)

Table S9a. ^{13}C and ^{1}H NMR data of protocatechuic acid 3-*O*-sulfate (399.87 MHz for ^{1}H, 100.55 MHz for ^{13}C, DMSO-d_6, 30 °C)

Atom	δ_C [ppm]	*m*	δ_H [ppm]	*n*(H)	*m*	*J* [HZ]
1	124.7^H	S	-	0	-	-
2	123.79	D	7.766	1	d	2.0
3	140.33	S	-	0	-	-
4	152.26	S	-	0	-	-
5	116.13	D	6.810	1	d	8.4
6	126.06	D	7.516	1	dd	8.4, 2.0
CO	168.16	S	-	0	-	-

δ - chemical shift, *m* – multiplicity, *n* – number of hydrogens, *J* – interaction constant, ^{H} HMBC readout

Table S9b. ^{13}C and ^{1}H NMR data of protocatechuic acid 4-*O*-sulfate (399.87 MHz for ^{1}H, 100.55 MHz for ^{13}C, DMSO-d_6, 30 °C)

Atom	δ_C [ppm]	*m*	δ_H [ppm]	*n*(H)	*m*	*J* [HZ]
1	n.d.	S	-	0	-	-
2	117.7^H	D	7.342	1	d	2.0
3	148.0^H	S	-	0	-	-
4	n.d.	S	-	0	-	-
5	121.3H	D	7.209	1	d	8.3
6	120.50	D	7.301	1	dd	8.3, 2.0
CO	n.d.	S	-	0	-	-

n.d. - not detected, ^{H} - HSQC/HMBC readout, δ - chemical shift, *m* – multiplicity, *n* – number of hydrogens, *J* – interaction constant

molar ratio 3-*O*-sulfate : 4-*O*-sulfate = 70 : 30

International Journal of
Molecular Sciences

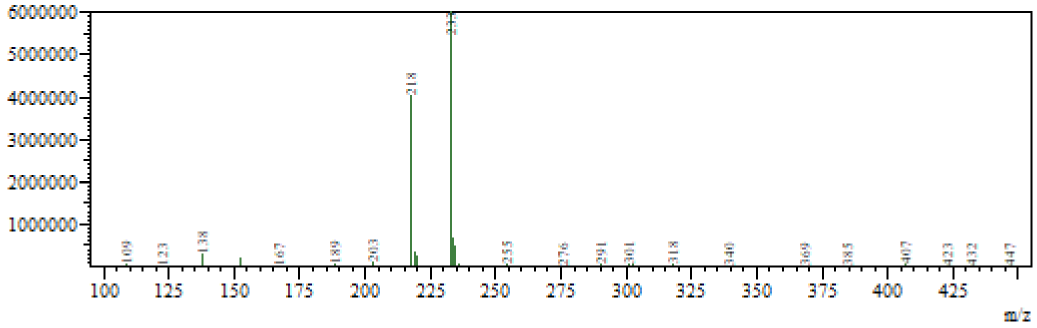

Figure S4. MS-ESI⁻ spectrum of protocatechuic acid *O*-sulfates ($[M - H]^-$, *m/z* calcd for $C_7H_5O_7S$: 232.98; found: 233). The peak *m/z* 218 belongs to *p*-nitrophenyl sulfate.

2.3 2,3,4-Trihydroxybenzoic acid O*-sulfates (THB-S)*

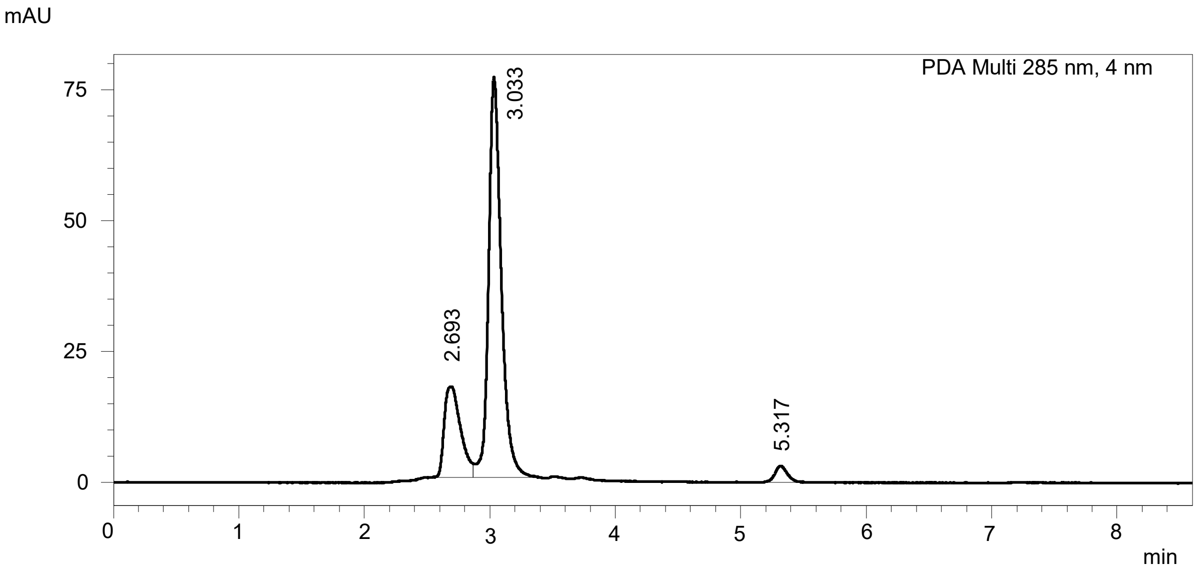

Figure S5. HPLC chromatogram of 2,3,4-trihydroxybenzoic acid *O*-sulfates (THB-S, regioisomers), method M1 (RT= 2.693 min and 3.033 min , 96% purity). The peak at 5.317 min belongs to *p*NP-S.

Table S10a. ^{13}C and ^{1}H NMR data of the major 2,3,4-trihydroxybenzoic acid *O*-sulfate[a] (600.23 MHz for ^{1}H, 150.93 MHz for ^{13}C, DMSO-d_6, 30 °C)

Atom	δ_C [ppm]	*m*	δ_H [ppm]	*n*(H)	*m*	*J* [HZ]
1	113.04	S	-	0	-	-
2	157.53	S	-	0	-	-
3	128.50	S	-	0	-	-
4	152.95	S	-	0	-	-
5	104.74	D	6.068	1	d	8.5

6	126.10	D	7.325	1	d	8.5
CO	172.01	S	-	0	-	-

Table S10b. ^{13}C and ^{1}H NMR data of the minor 2,3,4-trihydroxybenzoic acid *O*-sulfate[a] (600.23 MHz for ^{1}H, 150.93 MHz for ^{13}C, DMSO-d_6, 30 °C)

Atom	δ_C [ppm]	*m*	δ_H [ppm]	*n*(H)	*m*	*J* [HZ]
1	116.15	S	-	0	-	-
2	153.30	S	-	0	-	-
3	137.13	S	-	0	-	-
4	142.58	S	-	0	-	-
5	110.05	D	6.428	1	d	8.6
6	119.28	D	7.082	1	d	8.6
CO	171.93	S	-	0	-	-

[a] … the number and position of sulfate groups could not be unambiguously determined by NMR

molar ratio major sulfate : minor sulfate = 80 : 20

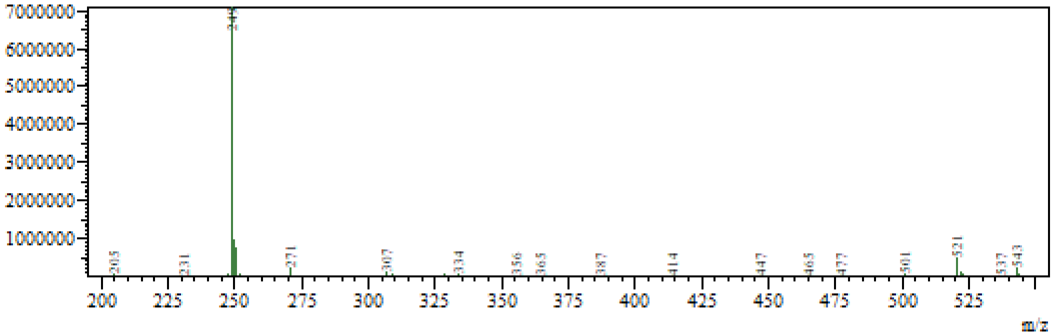

Figure S6. MS-ESI$^-$ spectrum of 2,3,4-trihydroxybenzoic acid *O*-sulfate([M - H]$^-$, *m/z* calcd for $C_7H_5O_8S$: 248.98; found: 249).

International Journal of
Molecular Sciences
MDPI

2.4 Caffeic acid O-sulfates (CAF-S)

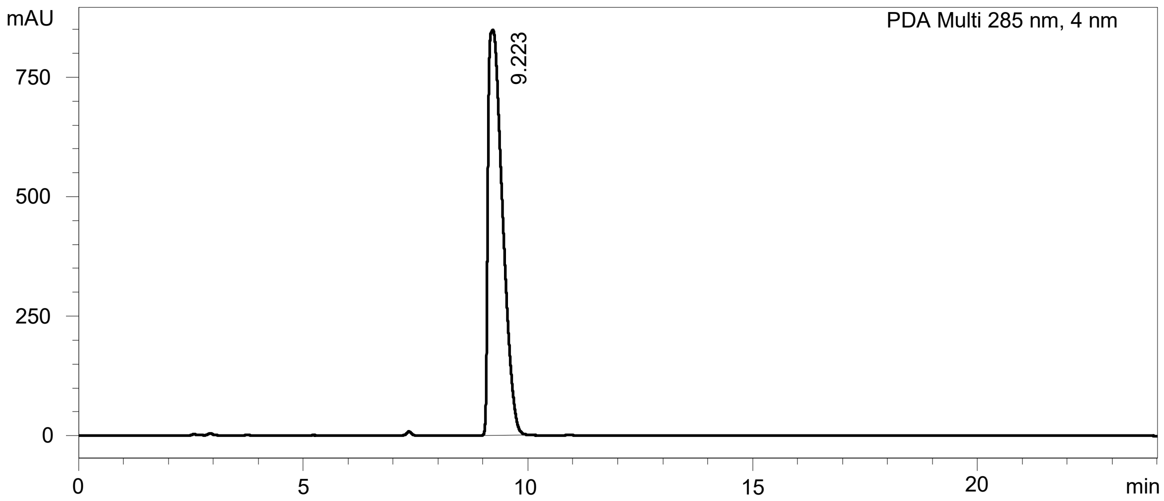

Figure S7. HPLC chromatogram of caffeic acid *O*-sulfates (CAF, regioisomers), method M2 (RT= 9.223 min, 99% purity)

Table S11a. ^{13}C and ^{1}H NMR data of caffeic acid 3-*O*-sulfate (600.23 MHz for ^{1}H, 150.93 MHz for ^{13}C, DMSO-d_6, 30 °C)

Atom	δ_C [ppm]	*m*	δ_H [ppm]	*n*(H)	*m*	*J* [HZ]
**1**	126.53	S	-	0	-	-
**2**	121.72	D	7.396	1	d	2.2
**3**	141.10	S	-	0	-	-
**4**	150.62	S	-	0	-	-
**5**	117.23	D	6.819	1	d	8.3
**6**	124.82	D	7.195	1	ddd	8.3, 2.2, 0.3
**1′**	169.39	S	-	0	-	-
**2′**	119.90	D	6.191	1	d	15.8
**3′**	140.84	D	7.291	1	d	15.8

δ - chemical shift, *m* – multiplicity, *n* – number of hydrogens, *J* – interaction constant

Table S11b. ^{13}C and ^{1}H NMR data of caffeic acid 4-*O*-sulfate (600.23 MHz for ^{1}H, 150.93 MHz for ^{13}C, DMSO-d_6, 30 °C)

Atom	δ_C [ppm]	*m*	δ_H [ppm]	*n*(H)	*m*	*J* [HZ]
**1**	131.67	S	-	0	-	-
**2**	115.72	D	7.028	1	d	2.2
**3**	149.00	S	-	0	-	-
**4**	142.08	S	-	0	-	-
**5**	122.65	D	7.162	1	d	8.3
**6**	119.19	D	6.969	1	ddd	8.3, 2.2, 0.3
**1′**	169.61	S	-	0	-	-

**2′**	122.54	D	6.270	1	d	15.8
**3′**	140.15	D	7.258	1	d	15.8

δ - chemical shift, *m* – multiplicity, *n* – number of hydrogens, *J* – interaction constant
ratio of 3-O-sulfate : 4-O-sulfate = 69 : 31

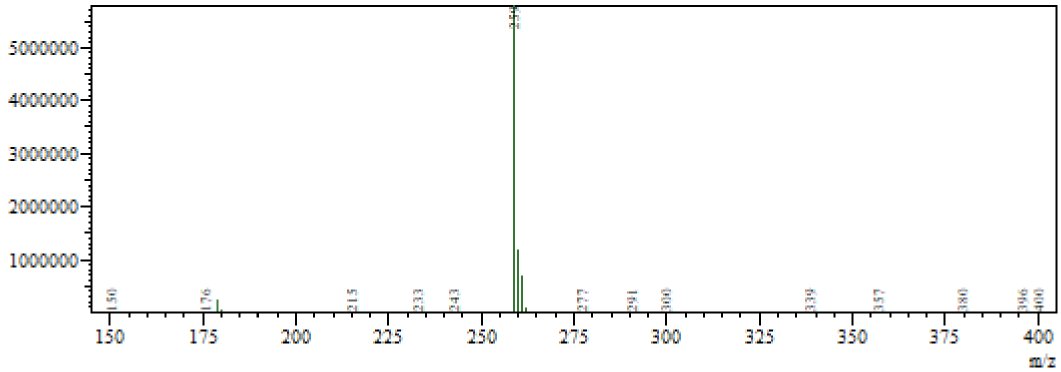

Figure S8. MS-ESI$^-$ spectrum of caffeic acid *O*-sulfates ([M - H]$^-$, *m/z* calcd for $C_9H_7O_7S$: 258.99; found: 259)

2.5 Catechol-1-O-sulfate (CAT-S)

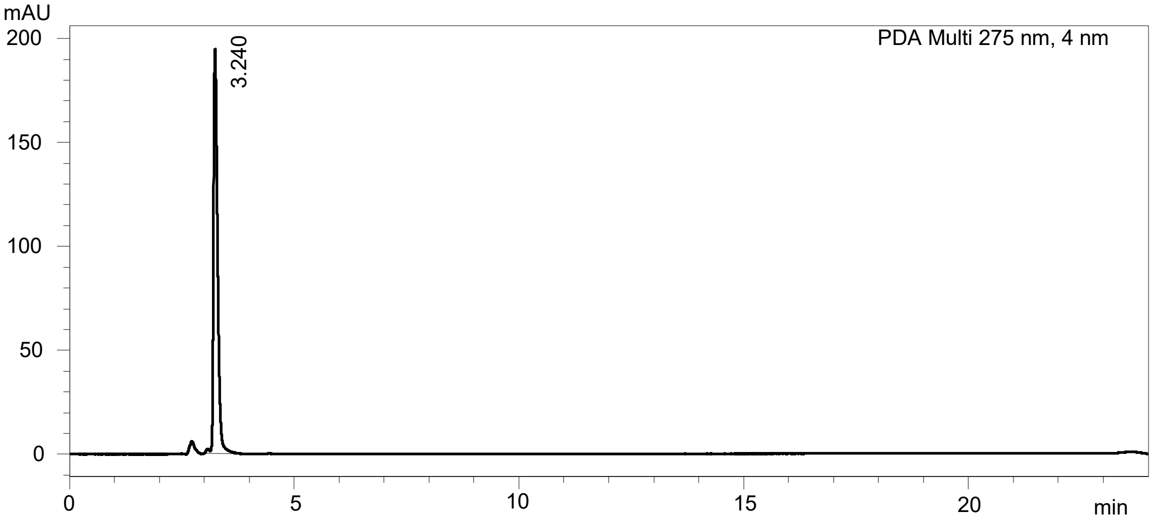

Figure S9. HPLC chromatogram of catechol-1-*O*-sulfate (Cat-S), method M1 (RT= 3.240 min, 96% purity)

Table S12. ^{13}C and ^{1}H NMR data of catechol-1-*O*-sulfate (399.87 MHz for ^{1}H, 100.55 MHz for ^{13}C, DMSO-*d*$_6$, 30 °C)

Atom	δ_C [ppm]	*m*	δ_H [ppm]	*n*(H)	*m*	*J* [HZ]
**1**	140.87	S	-	0	-	-
**2**	149.16	S	-	0	-	-
**3**	117.16	D	6.815	1	dd	8.0, 1.7
**4**	124.79	D	6.943	1	ddd	7.0, 7.3, 1.6

**5**	119.27	D	6.737	1	ddd	8.0, 7.3, 1.7
**6**	123.07	D	7.109	1	dd	8.0, 1.6

δ - chemical shift, *m* – multiplicity, *n* – number of hydrogens, *J* – interaction constant

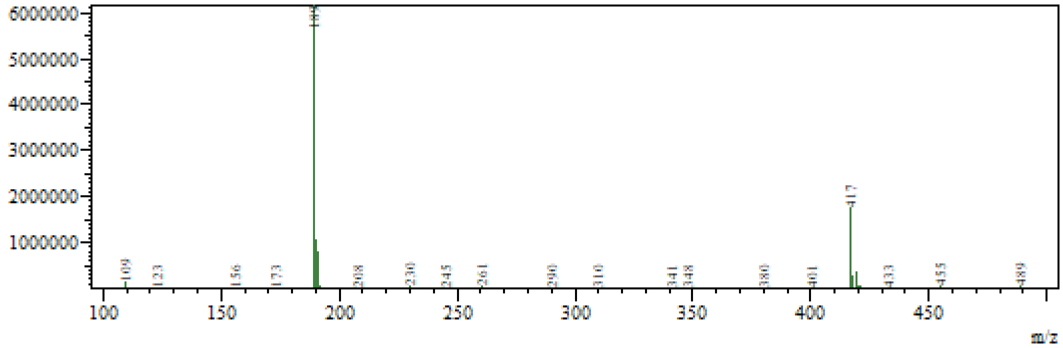

Figure S10. MS-ESI$^-$ spectrum of catechol-1-*O*-sulfate([M - H]$^-$, *m/z* calcd for $C_6H_5O_5S$: 188.99; found: 189)

2.6 Phloroglucinol-O-sulfate (PG-S)

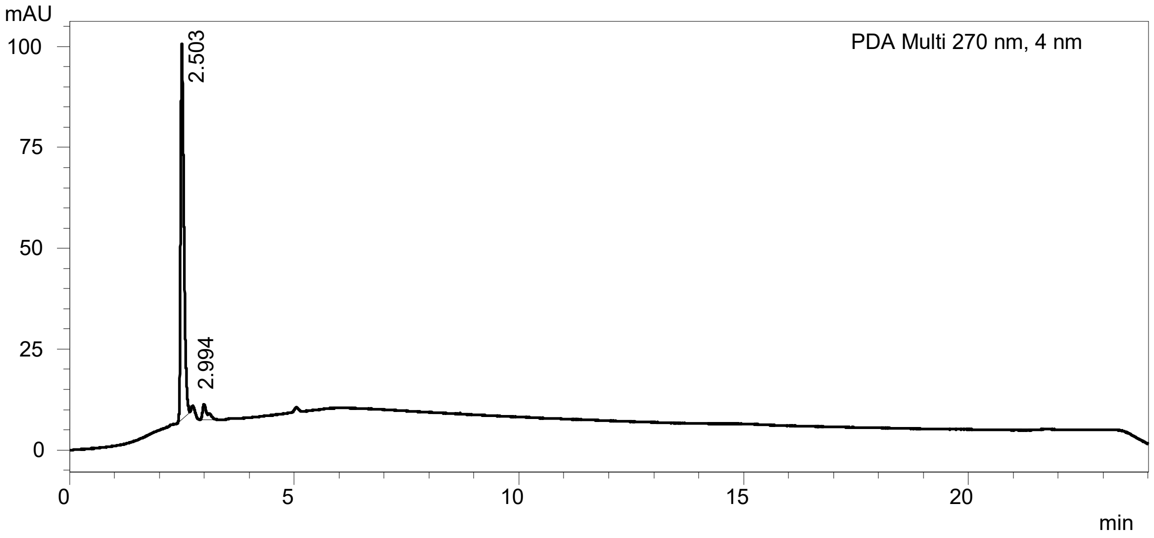

Figure S11. HPLC chromatogram of phloroglucinol-*O*-sulfate (PG-S), method M1 (RT= 2.503 min, 94% purity)

Table S13. ^{13}C and ^{1}H NMR data of phloroglucinol-*O*-sulfate (600.23 MHz for ^{1}H, 150.93 MHz for ^{13}C, DMSO-d_6, 30 °C)

Atom	δ_C [ppm]	*m*	δ_H [ppm]	*n*(H)	*m*	*J* [HZ]
1	154.87	S	-	0	-	-
2, 6	98.87	D	6.102	2	d	2.2

3, 5	158.00	S	-	0	-	-
4	97.50	D	5.881	1	t	2.2

δ - chemical shift, *m* – multiplicity, *n* – number of hydrogens, *J* – interaction constant

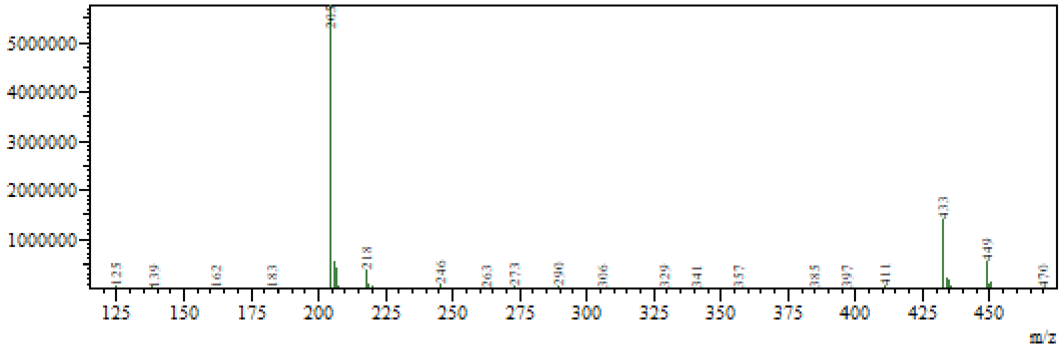

Figure S12. MS-ESI$^-$ spectrum of phloroglucinol-*O*-sulfate ([M - H]$^-$, *m/z* calcd for $C_6H_5O_6S$: 204.99; found: 205)

3 RETENTION TIMES, WIDTHS OF THE PEAKS AND ABSORPTION MAXIMA OF THE COMPOUNDS

3.1 Methods without buffer in the mobile phase

Table S1: Retention times (t_R), peak widths ($w_{0.05}$), and maximum absorption wavelengths (λ_{max}) for analytes measured separately by method **M3** (mobile phases: A= 0.1% TFA , B= 100% MeOH) , stationary phase PFP

Analyte	t_R [min]	$w_{0.05}$[a] [min]	λ_{max} [nm]
QUE	17.470	0.554	250, 264, 365
QUE-S	15.084	0.632	250, 264, 365
QUE-SS	11.084	1.703	250, 264, 365
AMP	6.062	0.314	291
AMP-S	7.449	0.437	291
LUT	20.010	0.422	268, 332
LUT-S	16.741	0.501	268, 332
LUT-SS	13.595	1.426	268, 332
MYR	14.014	0.410	253, 372
MYR-S	15.877	0.542	253, 372
MYR-SS	12.497	1.398	253, 372
MeCAT	16.713	0.582	276
MeCAT-S	12.519	0.738	276
ISQ	9.615	0.312	255, 354
ISQ-S	8.247	0.381	265, 337
RUT	9.156	0.322	256, 355
RUT-SS	7.175, 7.399[b]	n.d.[c]	266, 338
TAX	7.767	0.466	286
TAX-S	7.557	0.368	286
*p*NP	13.414	0.367	218, 314
*p*NP-S	6.751	0.295	281

[a] $w_{0.05}$ is the width of the peak in 5% of its height; [b] partial separation of sulfated regioisomers; [c] the peak shape did not allow the determination of $w_{0.05}$

Table S2: Retention times (t_R), peak widths ($w_{0.05}$), and maximum absorption wavelengths (λ_{max}) for analytes measured separately by method **M6** (mobile phases: A= 5% acetonitrile, 0.1% HCOOH, B= 80% acetonitrile, 0.1% HCOOH), stationary phase C18

Analyte	t_R [min]	$w_{0.05}$[a] [min]	λ_{max} [nm]
DHSCH	6.957	0.101	256, 374
DHSCH-S	6.771	0.478	256, 372
DHSB	7.039	0.177	256, 374
DHSB-S	6.771	0.608	254, 367
DHSB-SS	3.180	1.701	255, 369
SCH	5.753	0.156	286
SCH-S	4.985	0.393	286
SB	4.141, 4.225[b]	n.d.[c]	286
SB-S	3.165	0.981	286
CAF	3.087	0.211	217, 234, 324
CAF-S	5.847	2.555	215, 297, 307
CAT	2.317	0.246	225, 274
CAT-S	5.109	2.564	225, 267
PRO	1.822	0.237	230, 272, 296
PRO-S	4.697	2.579	220, 254
THB	2.058	0.259	255, 264
THB-S	0.718	0.050	255, 279
MeCAT	3.769	0.300	217, 277
MeCAT-S	6.578	2.546	218, 276
PG	1.039	0.197	269
PG-S	2.683	1.534	270
*p*NP	5.031	0.221	218, 314
*p*NP-S	0.796	0.140	281

[a] $w_{0.05}$ is the width of the peak in 5% of its height; [b] partial separation of stereoisomers A and B; [c] the peak shape did not allow the determination of $w_{0.05}$

International Journal of
Molecular Sciences

MDPI

Table S3: Retention times (t_R), peak widths ($w_{0.05}$), and maximum absorption wavelengths (λ_{max}) for analytes measured separately by method **M7** (mobile phases: A=5% acetonitrile, 0.1% HCOOH, mobile phase B= 80% AcN, 0.1% HCOOH), stationary phase C18-Polar

Analyte	t_R [min]	$w_{0.05}$[a] [min]	λ_{max} [nm]
DHSB	9.032	0.143	256, 374
DHSB-S	7.904	0.234	254, 367
DHSB-SS	8.490	0.146	255, 369
DHSCH	7.573	0.125	256, 374
DHSCH-S	6.768	0.322	256, 373
SCH	7.413	0.132	287
SCH-S	6.313	0.395	287
SB	7.410	0.186	287
SB-S	6.555	0.186	287
CAF	5.987	0.270	217, 234, 324
CAF-S	5.366	1.040	215, 231, 308
PRO	3.653	0.471	230, 272, 296
PRO-S	2.500	0.920	220, 254
THB	4.122	0.383	225, 264
THB-S	2.966	0.582	256, 294
CAT	4.331	0.403	273
CAT-S	7.627	0.305	273
MeCAT	6.302	0.261	220, 283
MeCAT-S	5.536	0.507	220, 275
PG	1.641	0.310	269
PG-S	1.265	0.308	270
*p*NP	7.274	0,278	218, 314
*p*NP-S	5.567	0.621	281

[a] $w_{0.05}$ is the width of the peak in 5% of its height

3.2 Methods with ammonium acetate buffer in the mobile phase

Table S4: Retention times (t_R), peak widths ($w_{0.05}$), and maximum absorption wavelengths (λ_{max}) for analytes measured separately by method **M1** (mobile phases: A= 10 mM ammonium acetate, 0.1% HCOOH, mobile phase B= 100% MeOH), stationary phase PFP

Analyte	t_R [min]	$w_{0.05}$[a] [min]	λ_{max} [nm]
QUE	16.127	0.385	251, 264, 360
QUE-S	12.139	0.397	251, 264, 362
QUE-SS	6.014	0.642	249, 265, 366
AMP	5.480	0.519	290
AMP-S	6.007	0.352	290
LUT	17.916	0.427	254, 349
LUT-S	13.340	0.377	268, 330
LUT-SS	12.830	0.362	268, 336
MYR	12.716	0.390	253, 316,369
MYR-S	8.566	0.478	267, 316, 357
MYR-SS	4.602	0.400	253, 316, 371
CAT	4.578	0.256	212, 276
CAT-S	3.240	0.207	212, 271
MeCAT	5.978	0.335	275
MeCAT-S	4.112	0.249	275
DHSB	19.094	1.030	254, 366
DHSB-S	17.385	0.347	255, 369
DHSB-SS	8.280	0.525	254, 347
DHSCH	17.010	0.425	256, 373
DHSCH-S	12.060	0.576	256, 370
SB	15.202, 15.521[c]	n.d.[b]	288
SB-S	13.728, 14.479 [d]	0.287, 0.415	
SCH	12.449, 13.285[c]	0.299, 0.312	288
SCH-S	6.176, 6.654[d]	0.338	288
CAF	5.150	0.238	217, 234, 324
CAF-S	3.720	0.267	213, 227, 294
PRO	3.721	0.270	262, 296
PRO-S	2.990	0.225	250
THB	3.568	0.246	261
THB-S	2.693, 3.033[d]	0.329, 0.266	252, 293
PG	2.906	0.205	299
PG-S	2.503	0.177	271
ISQ	9.397	0.344	256, 355
ISQ-S	6.681	0.311	266, 337
RUT	8.869	0.342	256, 355
RUT-SS	6.055	0.261	266, 338
TAX	7.380	0.285	288
TAX-S	5.582	0.321	289
*p*NP	11.528	0.338	218, 314

Analyte	t_R [min]	$w_{0.05}$[a] [min]	λ_{max} [nm]
*p*NP-S	4.930	0.457	280

[a] $w_{0.05}$ is the width of the peak in 5% of its height; [b]n.d. means that the peak shape did not allow the determination of $w_{0.05}$; [c] partial separation of stereoisomers A and B; [d] partial separation of sulfated regioisomers

Table S5: Retention times (t_R), peak widths ($w_{0.05}$), and maximum absorption wavelengths (λ_{max}) for analytes measured separately by method **M2** (mobile phases: A= 10 mM ammonium acetate, 0.1% HCOOH, mobile phase B= 100% MeOH), stationary phase PFP

Analyte	t_R [min]	$w_{0.05}$[a] [min]	λ_{max} [nm]
CAF	12.527	0.356	216, 235, 323
CAF-S	9.233	0.671	216, 294, 308
CAT	7.453	0.350	215, 273
CAT-S	4.938	0.386	215, 270
PRO	7.213	0.337	257, 293
PRO-S	5.541	0.403	220, 254
THB	6.240	0.368	260
THB-S	4.949	0.317	252, 293
MeCAT	11.944	0.508	278
MeCAT-S	7.910	0.524	276
PG	4.004	0.235	266
PG-S	3.339	0.266	270
*p*NP	20.542	0.385	218, 314
*p*NP-S	10.401	0.498	280

[a] $w_{0.05}$ is the width of the peak in 5% of its height

Table S6: Retention times (t_R), peak widths ($w_{0.05}$), and maximum absorption wavelengths (λ_{max}) for analytes measured separately by method **M5** (mobile phases: A= 10 mM ammonium acetate, 0.1% HCOOH, mobile phase B= 100% MeOH), stationary phase C18

**Analyte**	**t_R [min]**	**$w_{0.05}$[a] [min]**	**λ_{max} [nm]**
DHSB	7.293	0.146	254, 368
DHSB-S	6.914	0.189	254, 368
DHSB-SS	6.097	0.392	254, 346
DHSCH	6.691	0.108	256, 374
DHSCH-S	6.258	0.177	256, 346
SCH	6.002	n.d.[b]	287
SCH-S	5.552	n.d.[b]	287
SB	6.480, 6.514[c]	n.d.[b]	287
SB-S	6.487, 6.579[f]	n.d.[b]	287
CAF	4.954	0.102	218, 235, 323
CAF-S	4.326, 4.494[d]	n.d.[b]	229, 293
CAT	2.920	0.356	273
CAT-S	4.290	n.d.[b]	279
PRO	2.816	0.410	226, 258, 292
PRO-S	2.104	0.276	254
THB	1.944	0.175	231, 257
THB-S	n.d.[e]	-	252, 293
MeCAT	4.946	0.226	221, 274
MeCAT-S	4.362[d]	n.d.[b]	221, 274
PG	1.180	0.288	266
PG-S	1.465	0.567	276
*p*NP	5.609	0.169	218, 314
*p*NP-S	5.216	0.900	280

[a] $w_{0.05}$ is the width of the peak in 5% of its height; [b]n.d. means, that the peak shape did not allow the determination of $w_{0.05}$; [c] partial separation of stereoisomers A and B; [d] partial separation of sulfated regioisomers; [e] the compound was decomposed during the analysis, only parent compound without sulfate was detected; [f] separation of sulfated stereoisomers A and B

Table S7: Retention times (t_R), peak widths ($w_{0.05}$), and maximum absorption wavelengths (λ_{max}) for analytes measured separately by method **M4** (mobile phases: A= 10 mM ammonium acetate, 0.1% HCOOH, mobile phase B= 100% MeOH), stationary phase ZICpHILIC

Analyte	t_R [min]	$w_{0.05}$[a] [min]	λ_{max} [nm]
DHSB	1.160	0.251	255, 360
DHSB-S	3.636	0.849	254, 367
DHSB-SS	8.853	0.588	253, 346
DHSCH	2.025	1.209	256, 373
DHSCH-S	5.448	0.465	256, 373
SCH	3.047	1.210	287
SCH-S	6.332	0.540	286
SB	1.094	0.647	287
SB-S	2.470, 3.007, 4.365[c]	n.d.[b]	
CAF	2.540	0.660	217, 239, 295, 322
CAF-S	4.194	0.528	213, 295, 307
CAT	1.257	1.348	230, 273
CAT-S	n.d.[d]	-	
PRO	3.184	0.787	254, 291
PRO-S	6.255	0.558	253
THB	4.456	0.638	231, 265
THB-S	n.d.[d]	-	
MeCAT	0.963	n.d.[b]	221, 255
MeCAT-S	1.049	0.314	217, 293
PG	4.641	0.430	268
PG-S	9.500	0.732	271
*p*NP	0.984	0.273	218, 312
*p*NP-S	1.494	0.600	280

[a] $w_{0.05}$ is the width of the peak in 5% of its height; [b]n.d. means, that the peak shape did not allow the determination of $w_{0.05}$; [c] partial separation of sulfated stereoisomers; [d] the compound was decomposed during the analysis, only parent compound without sulfate was detected

International Journal of
Molecular Sciences

4 COMPARISON OF HPLC CHROMATOGRAMS FOR INDIVIDUAL COMPOUNDS

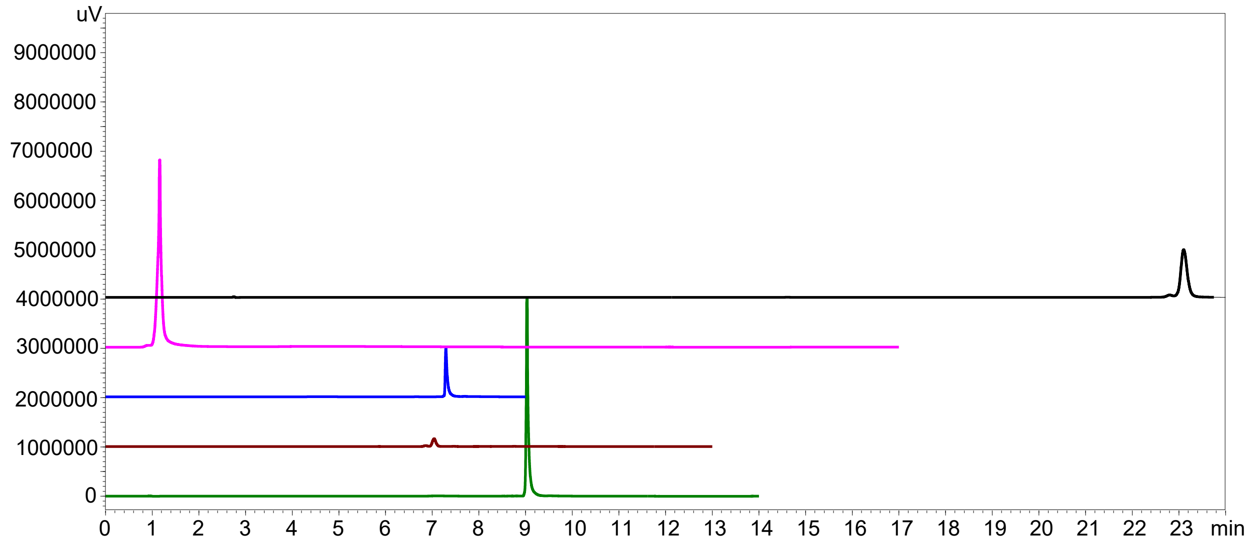

Figure S13: Comparison of HPLC chromatograms of **DHSB** in various methods (λ_{max} = 360 nm). Method M1 in black, method M4 in pink, method M5 in blue, method M6 in brown, method M7 in green.

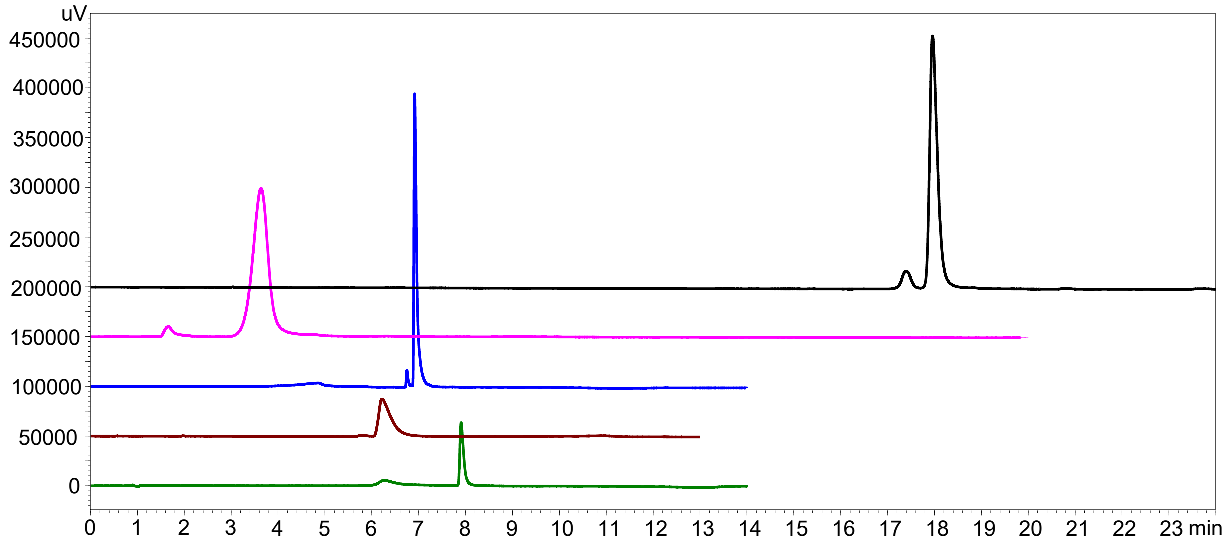

Figure S14: Comparison of HPLC chromatograms of **DHSB-S** in various methods (λ_{max} = 360 nm). Method M1 in black, method M4 in pink, method M5 in blue, method M6 in brown, method M7 in green.

International Journal of
Molecular Sciences

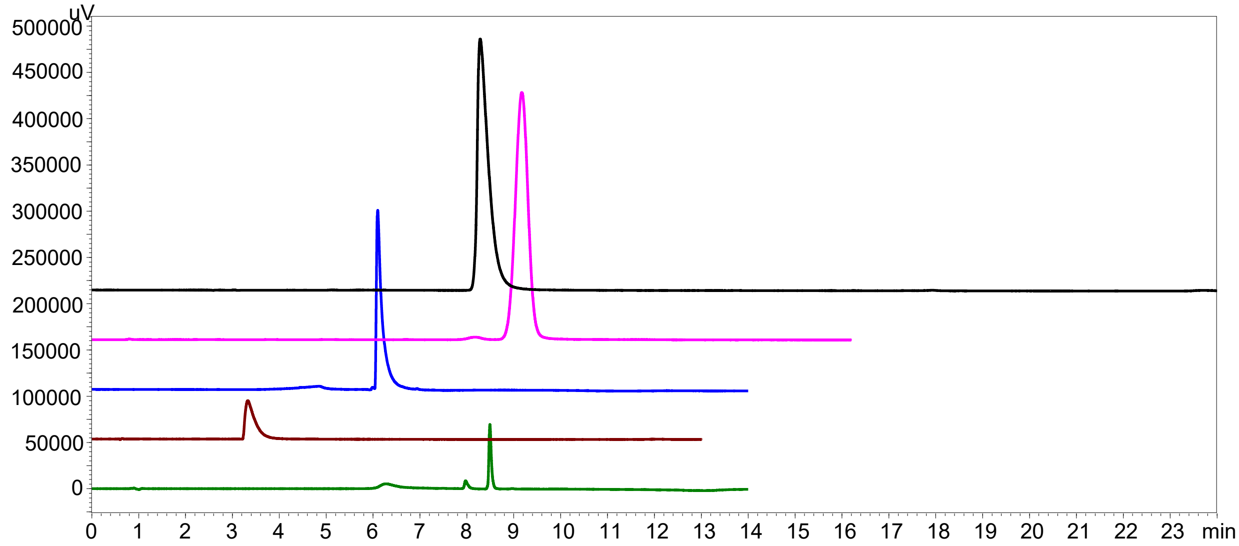

Figure S15: Comparison of HPLC chromatograms of **DHSB-SS** in various methods (λ_{max} = 360 nm). Method M1 in black, method M4 in pink, method M5 in blue, method M6 in brown, method M7 in green.

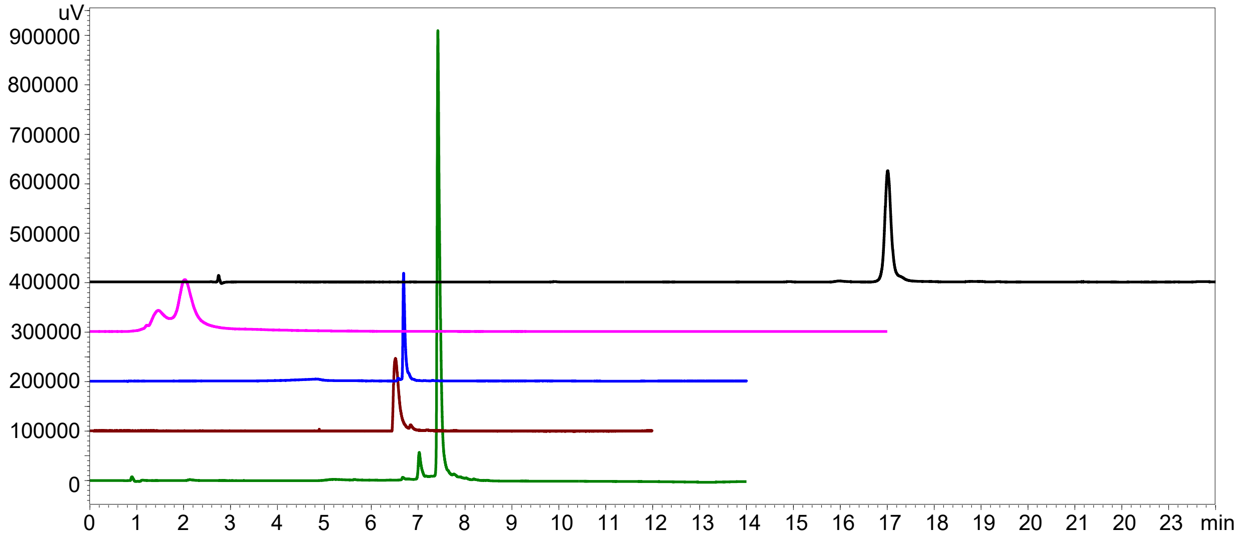

Figure S16: Comparison of HPLC chromatograms of **DHSCH** in various methods (λ_{max} = 360 nm). Method M1 in black, method M4 in pink, method M5 in blue, method M6 in brown, method M7 in green.

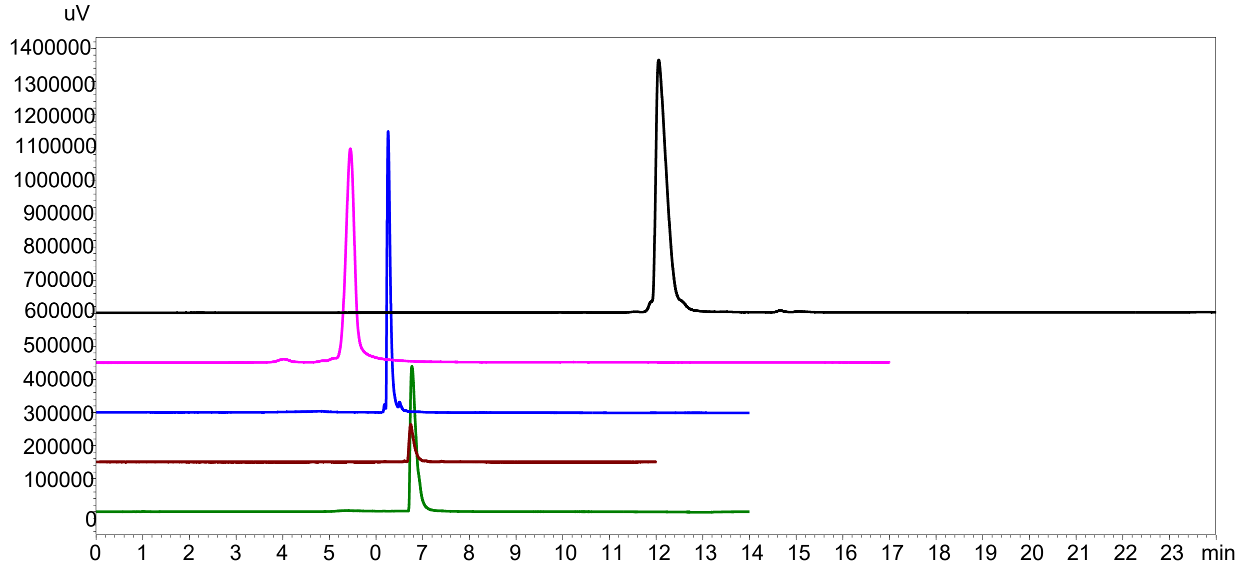

Figure S17: Comparison of HPLC chromatograms of **DHSCH-S** in various methods (λ_{max} = 360 nm). Method M1 in black, method M4 in pink, method M5 in blue, method M6 in brown, method M7 in green.

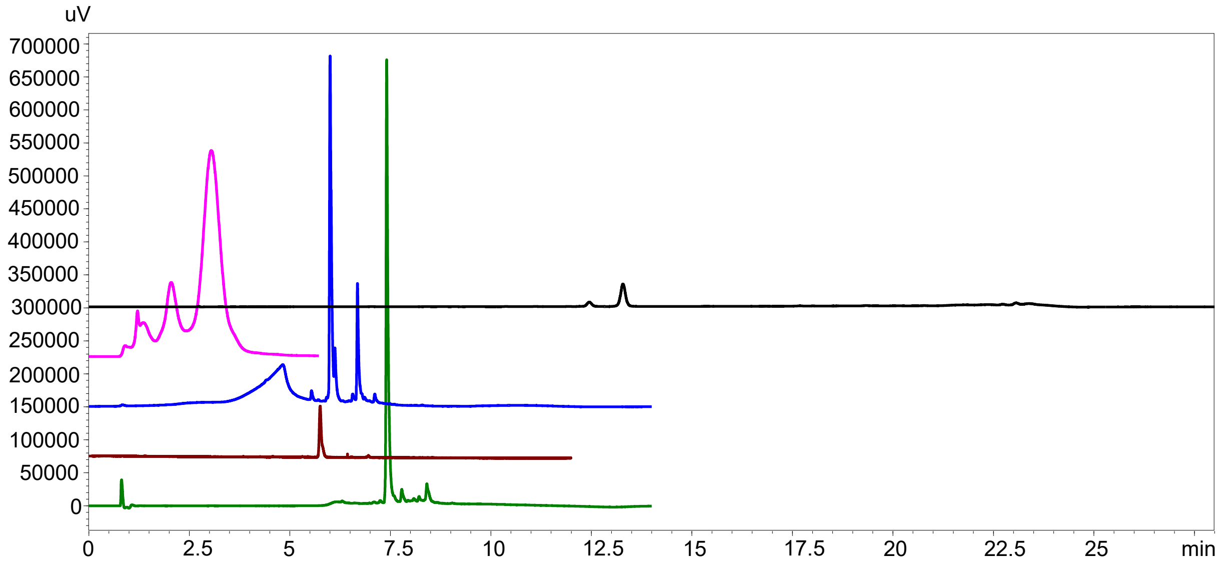

Figure S18: Comparison of HPLC chromatograms of **SCH** in various methods (λ_{max} = 285 nm). Method M1 in black, method M4 in pink, method M5 in blue, method M6 in brown, method M7 in green.

International Journal of
Molecular Sciences

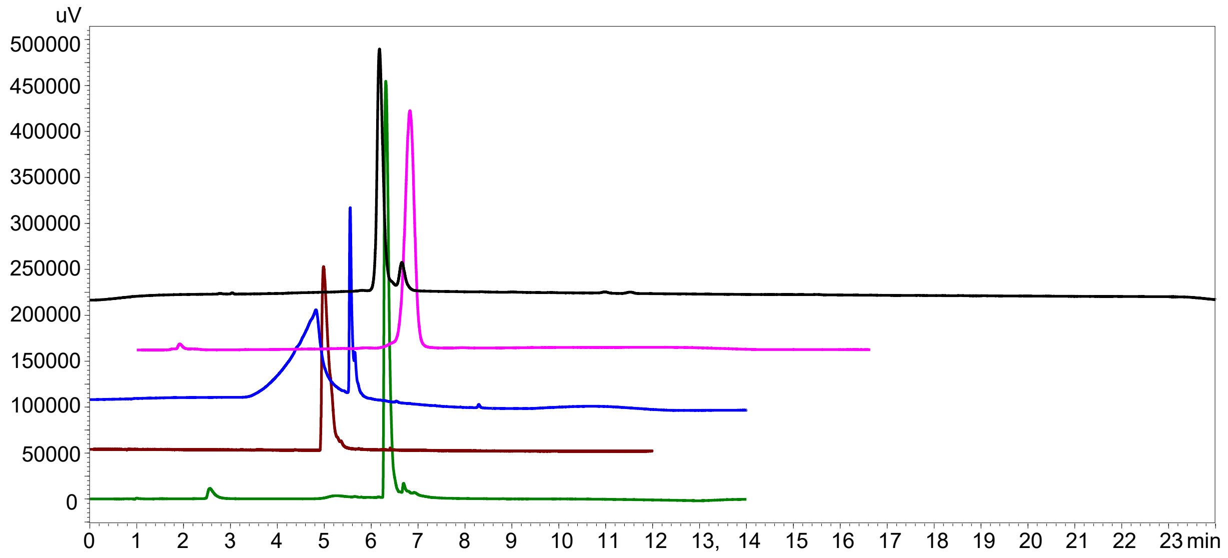

Figure S19: Comparison of HPLC chromatograms of **SCH-S** in various methods (λ_{max} = 285 nm). Method M1 in black, method M4 in pink, method M5 in blue, method M6 in brown, method M7 in green. Pink chromatogram is shifted by one minute for greater clarity.

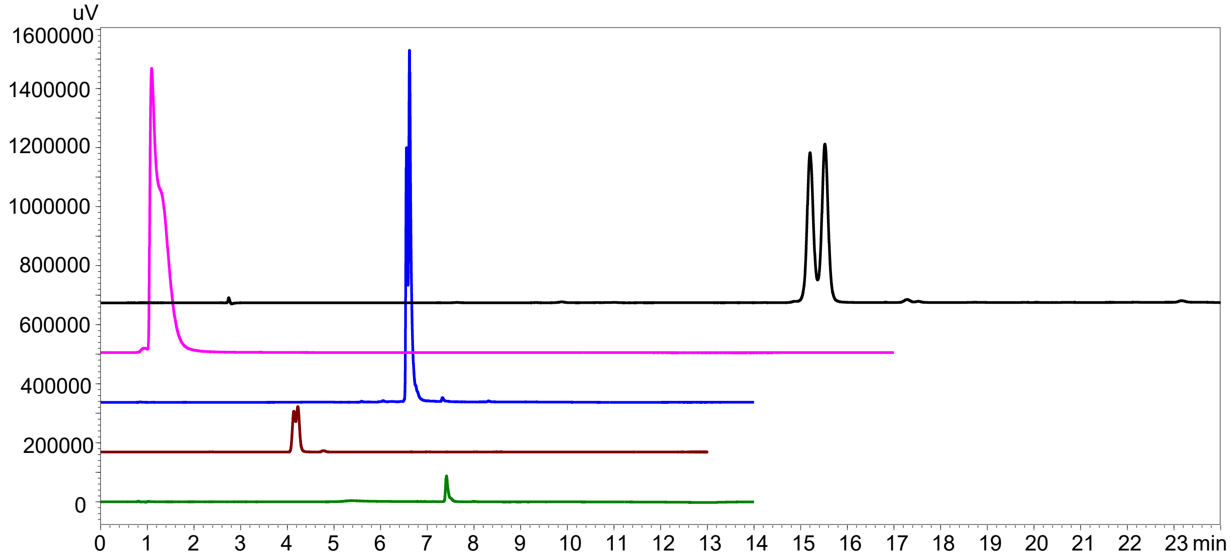

Figure S20: Comparison of HPLC chromatograms of **SB** in various methods (λ_{max} = 285 nm). Method M1 in black, method M4 in pink, method M5 in blue, method M6 in brown, method M7 in green.

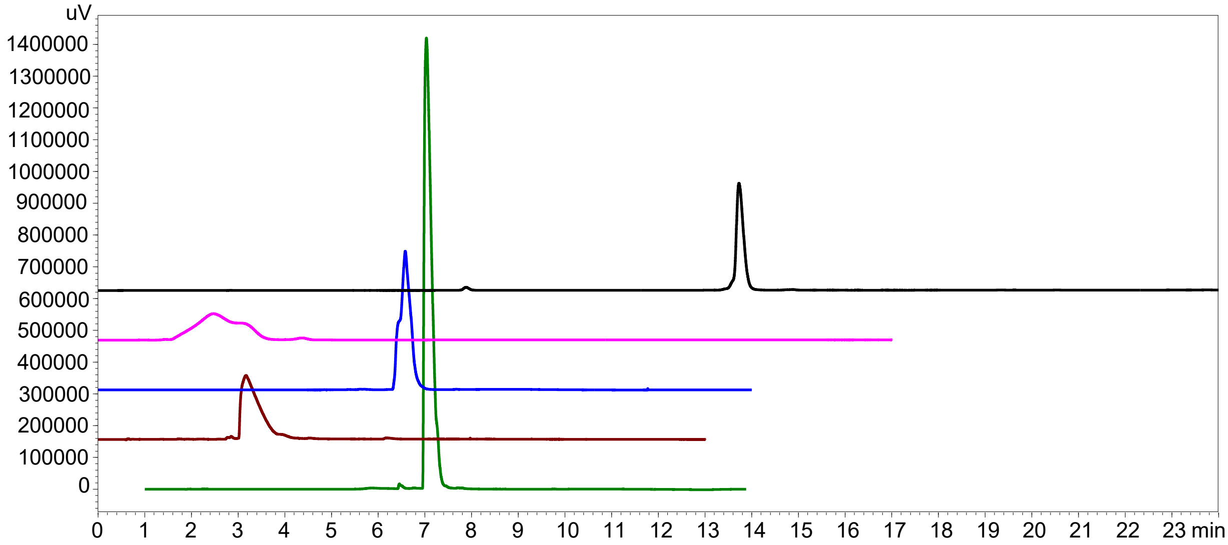

Figure S21: Comparison of HPLC chromatograms of **SB-S** in various methods (λ_{max} = 285 nm). Method M1 in black, method M4 in pink, method M5 in blue, method M6 in brown, method M7 in green. Green chromatogram is shifted by one minute for greater clarity.

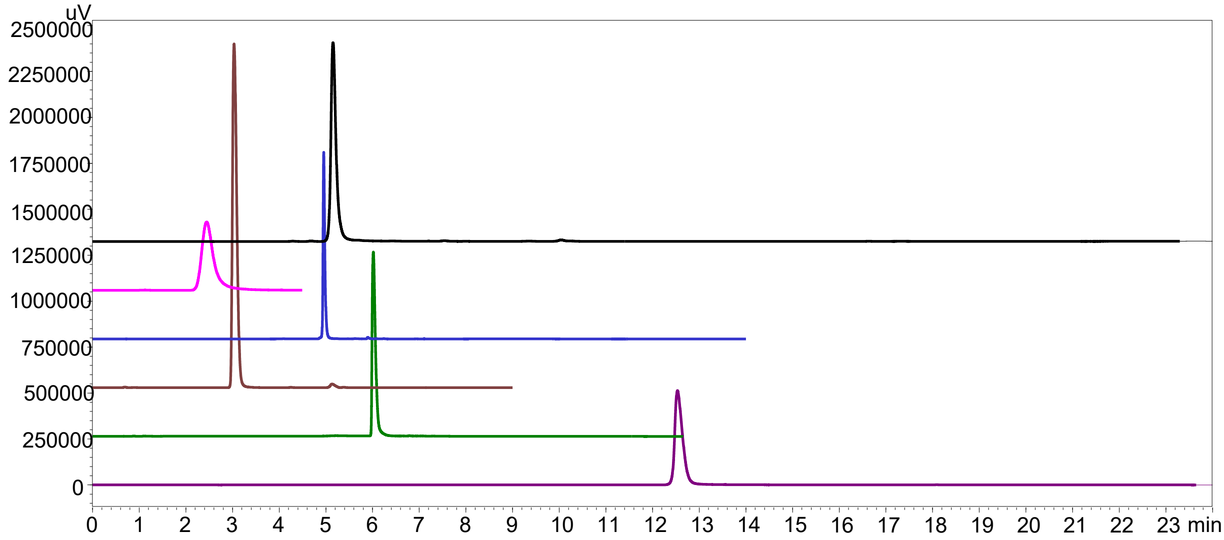

Figure S22: Comparison of HPLC chromatograms of **CAF** in various methods (λ_{max} = 324 nm). Method M1 in black, method M2 in purple, method M4 in pink, method M5 in blue, method M6 in brown, method M7 in green.

International Journal of
Molecular Sciences

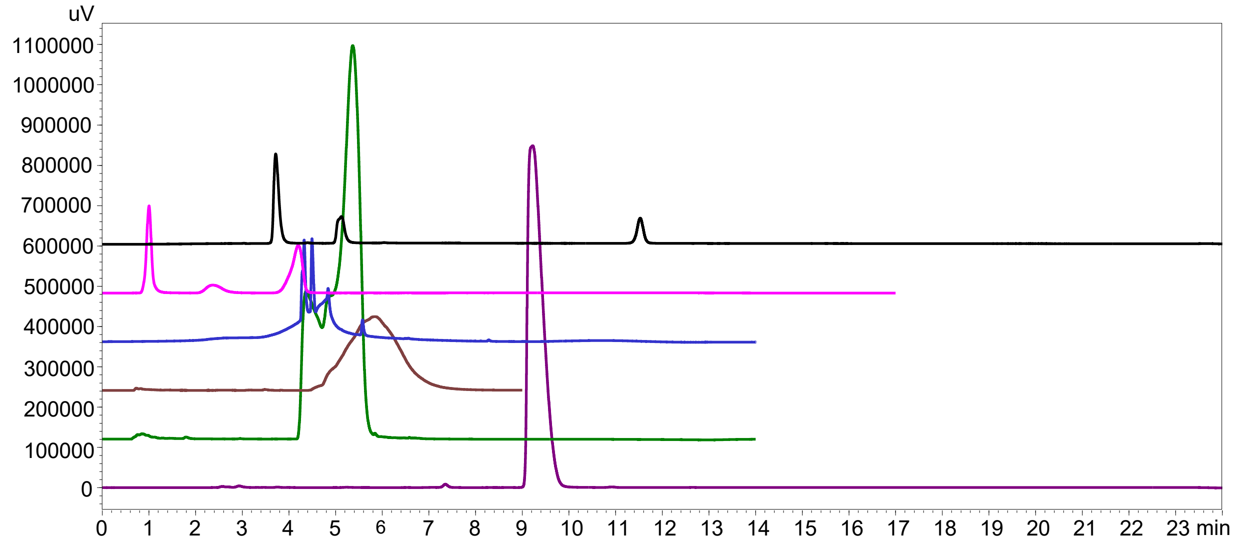

Figure S23: Comparison of HPLC chromatograms of **CAF-S** in various methods (λ_{max} = 285 nm). Method M1 in black, method M2 in purple, method M4 in pink, method M5 in blue, method M6 in brown, method M7 in green.

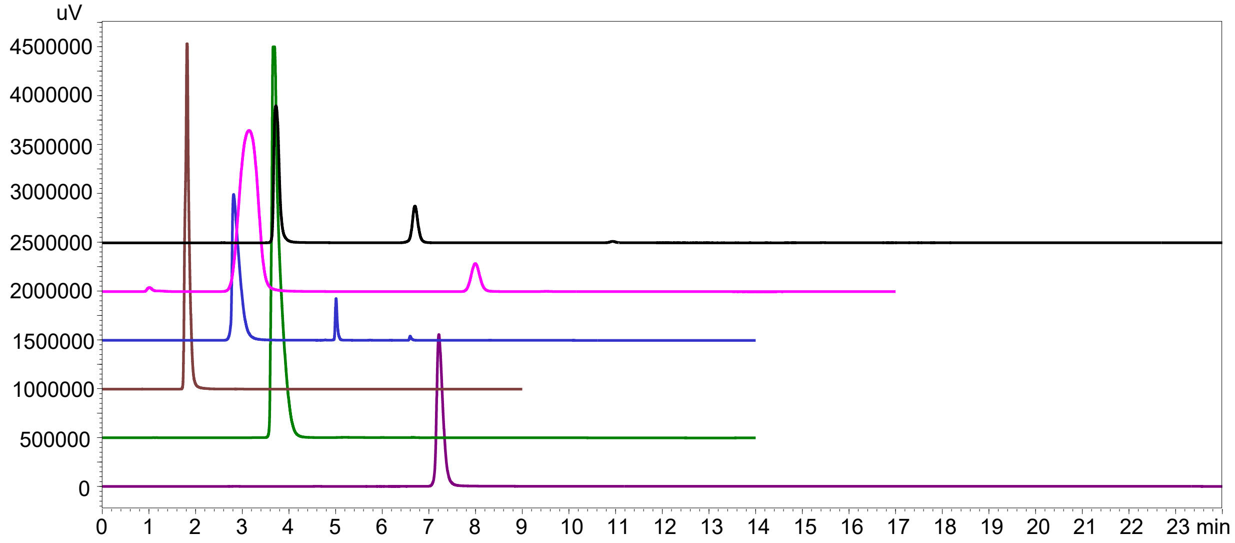

Figure S24: Comparison of HPLC chromatograms of **PRO** in various methods (λ_{max} = 285 nm). Method M1 in black, method M2 in purple, method M4 in pink, method M5 in blue, method M6 in brown, method M7 in green.

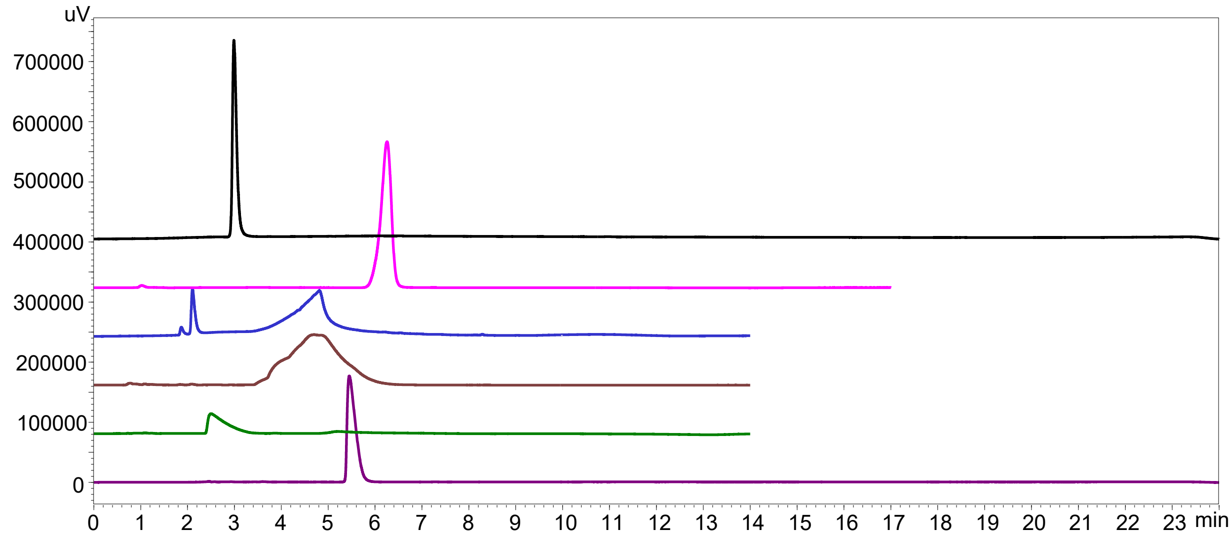

Figure S25: Comparison of HPLC chromatograms of **PRO-S** in various methods (λ_{max} = 250 nm). Method M1 in black, method M2 in purple, method M4 in pink, method M5 in blue, method M6 in brown, method M7 in green.

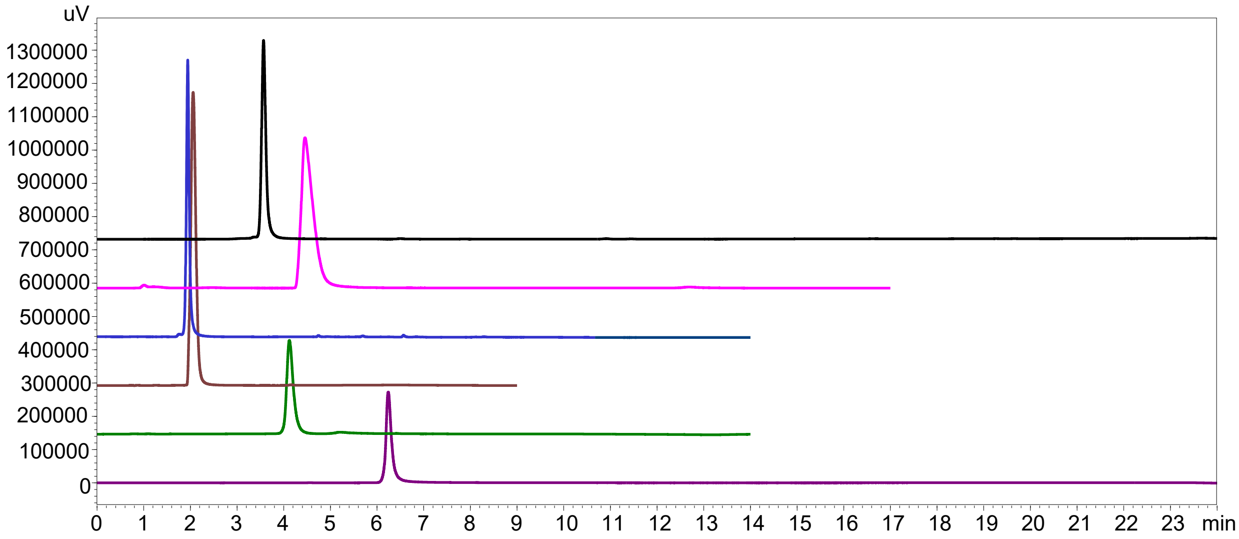

Figure S26: Comparison of HPLC chromatograms of **THB** in various methods (λ_{max} = 260 nm). Method M1 in black, method M2 in purple, method M4 in pink, method M5 in blue, method M6 in brown, method M7 in green.

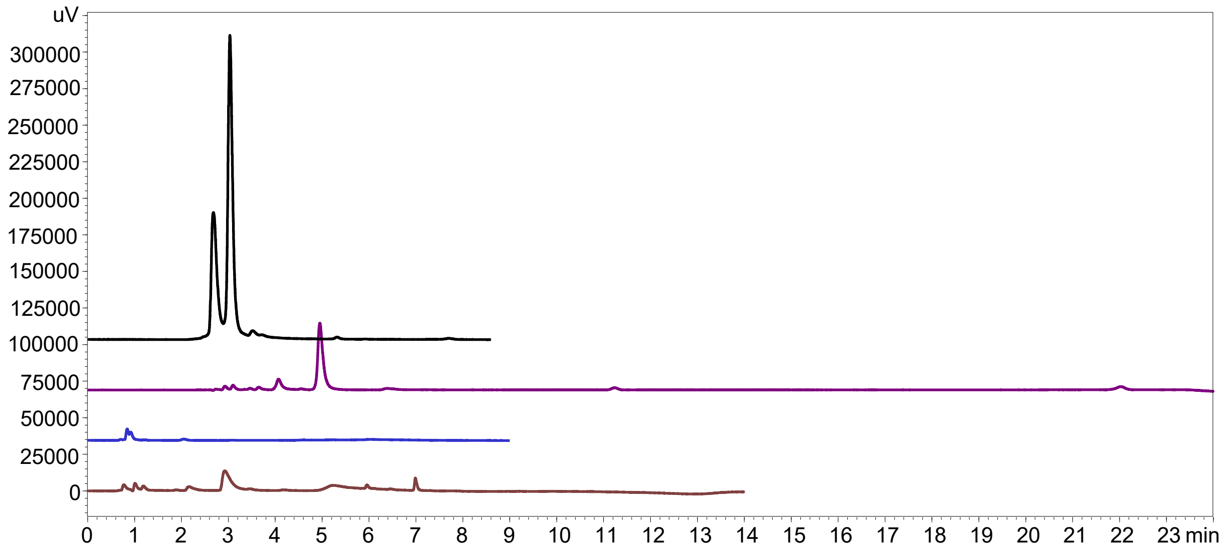

Figure S27: Comparison of HPLC chromatograms of **THB-S** in various methods (λ_{max} = 250 nm). Method M1 in black, method M2 in purple, method M4 in pink, method M5 in blue, method M6 in brown.

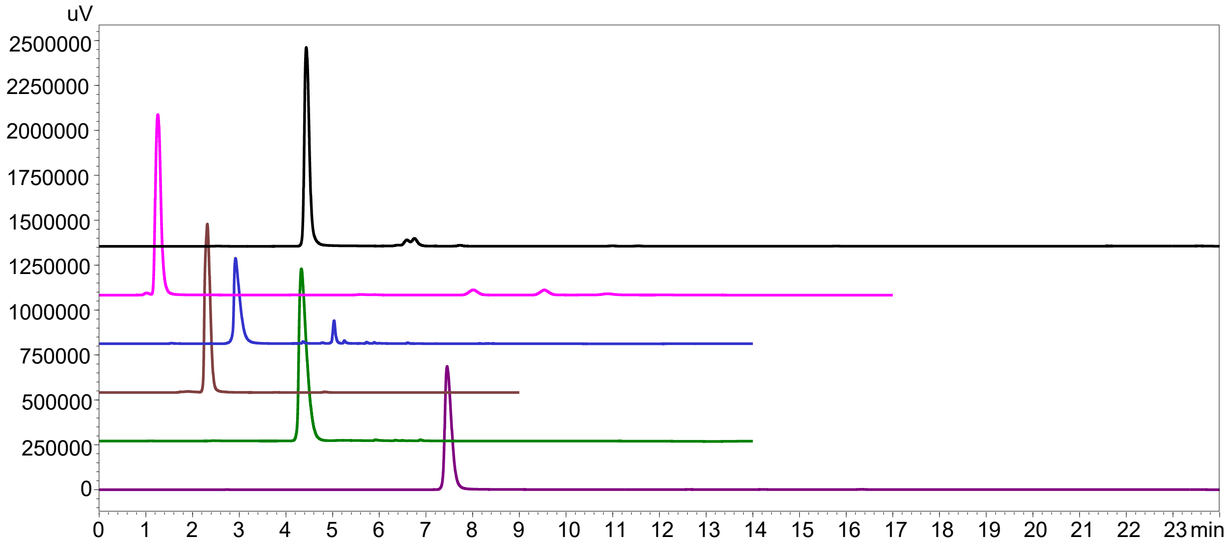

Figure S28: Comparison of HPLC chromatograms of **CAT** in various methods (λ_{max} = 270 nm). Method M1 in black, method M2 in purple, method M4 in pink, method M5 in blue, method M6 in brown, method M7 in green.

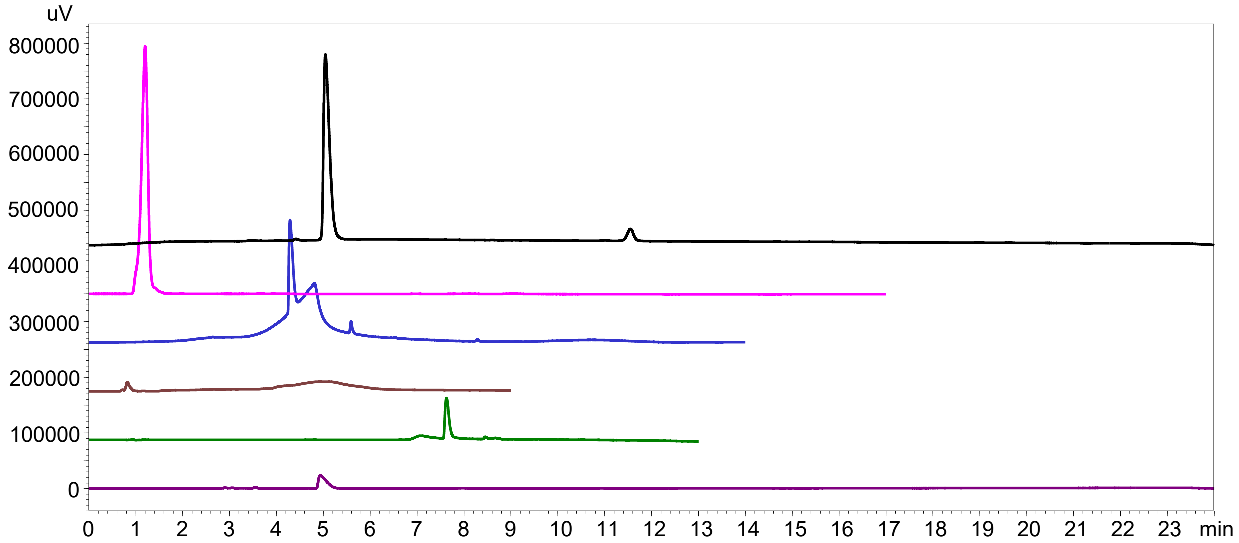

Figure S29: Comparison of HPLC chromatograms of **CAT-S** in various methods (λ_{max} = 285 nm). Method M1 in black, method M2 in purple, method M4 in pink, method M5 in blue, method M6 in brown, method M7 in green.

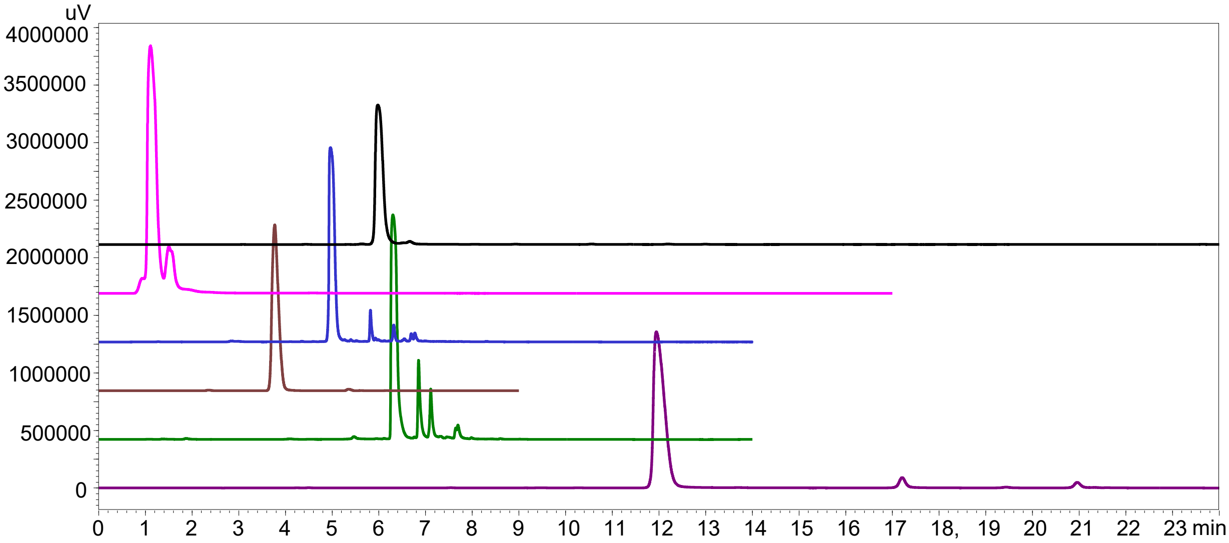

Figure S30: Comparison of HPLC chromatograms of **MeCAT** in various methods (λ_{max} = 285 nm). Method M1 in black, method M2 in purple, method M4 in pink, method M5 in blue, method M6 in brown, method M7 in green.

International Journal of
Molecular Sciences

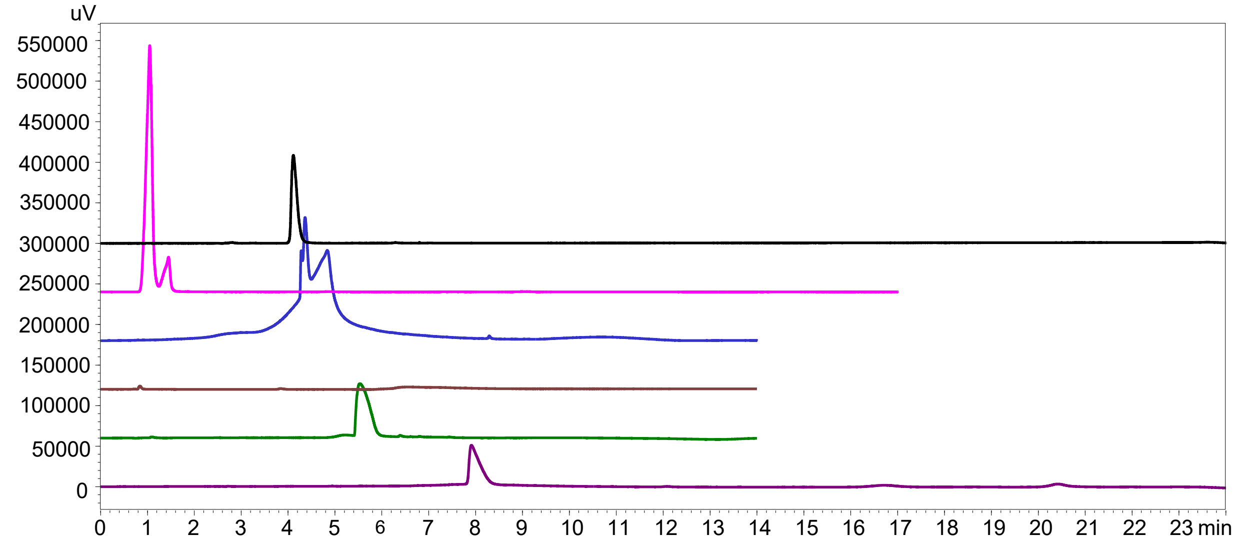

Figure S31: Comparison of HPLC chromatograms of **MeCAT-S** in various methods (λ_{max} = 285 nm). Method M1 in black, method M2 in purple, method M4 in pink, method M5 in blue, method M6 in brown, method M7 in green.

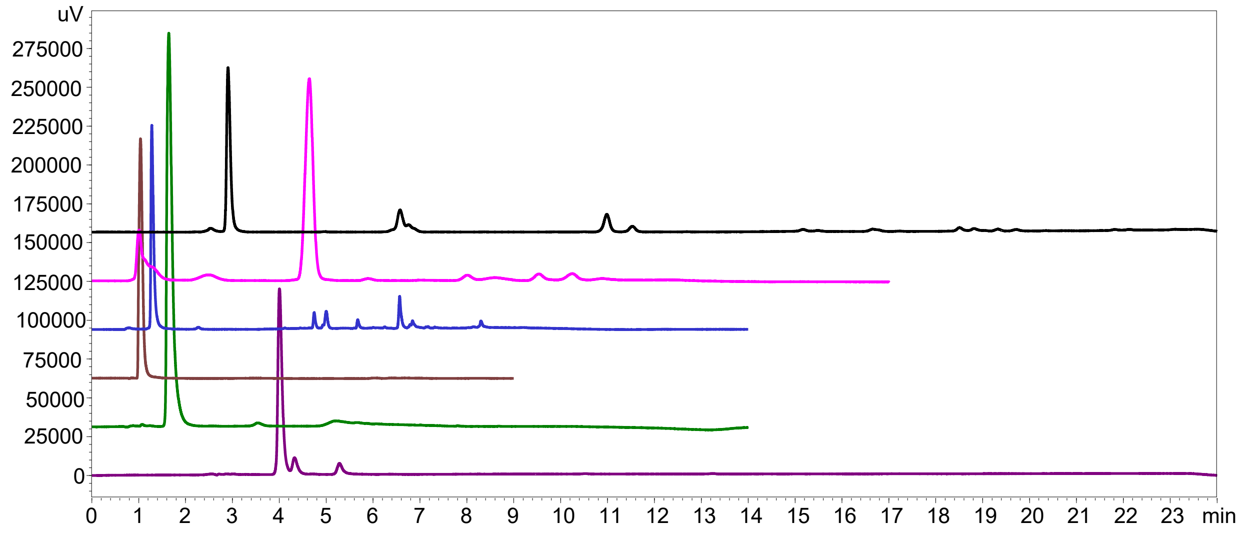

Figure S32: Comparison of HPLC chromatograms of **PG** in various methods (λ_{max} = 270 nm). Method M1 in black, method M2 in purple, method M4 in pink, method M5 in blue, method M6 in brown, method M7 in green.

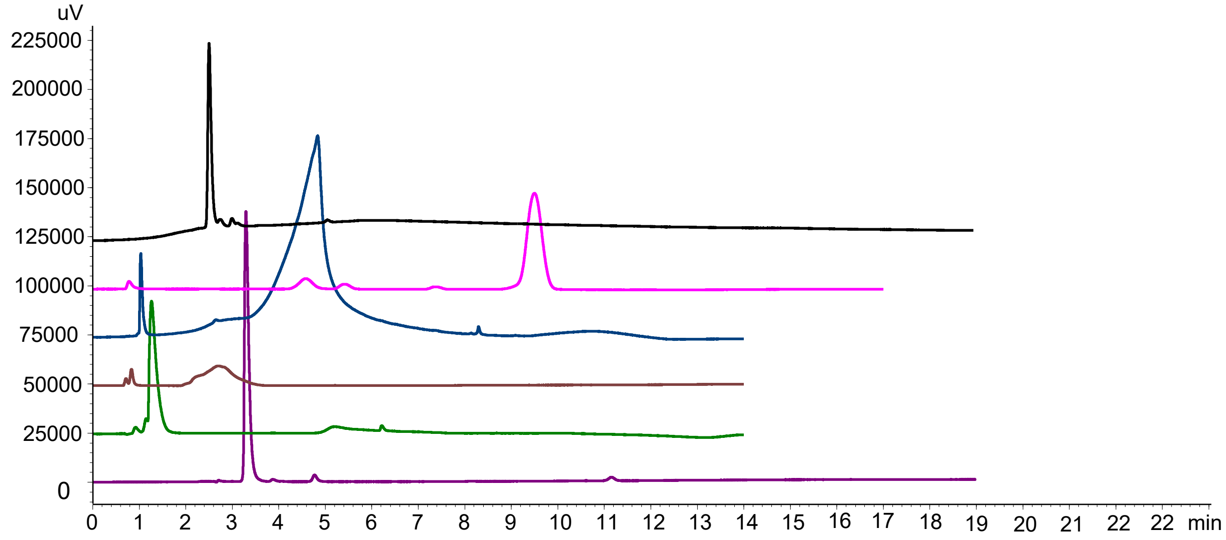

Figure S33: Comparison of HPLC chromatograms of **PG-S** in various methods (λ_{max} = 270 nm). Method M1 in black, method M2 in purple, method M4 in pink, method M5 in blue, method M6 in brown, method M7 in green.

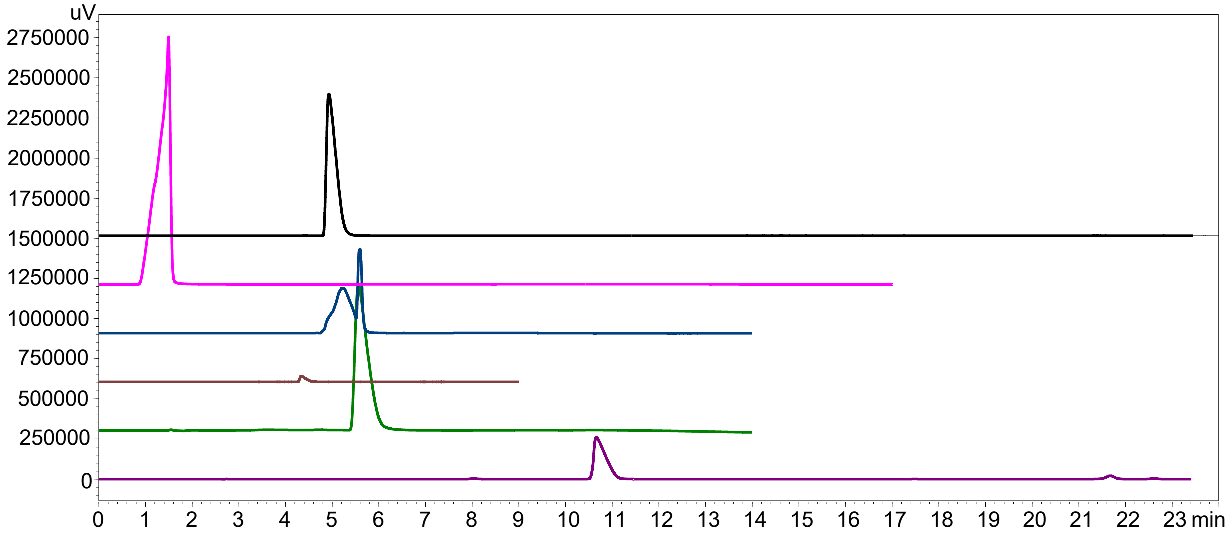

Figure S34: Comparison of HPLC chromatograms of ***p*NP-S** in various methods (λ_{max} = 280 nm). Method M1 in black, method M2 in purple, method M4 in pink, method M5 in blue, method M6 in brown, method M7 in green.

International Journal of
Molecular Sciences

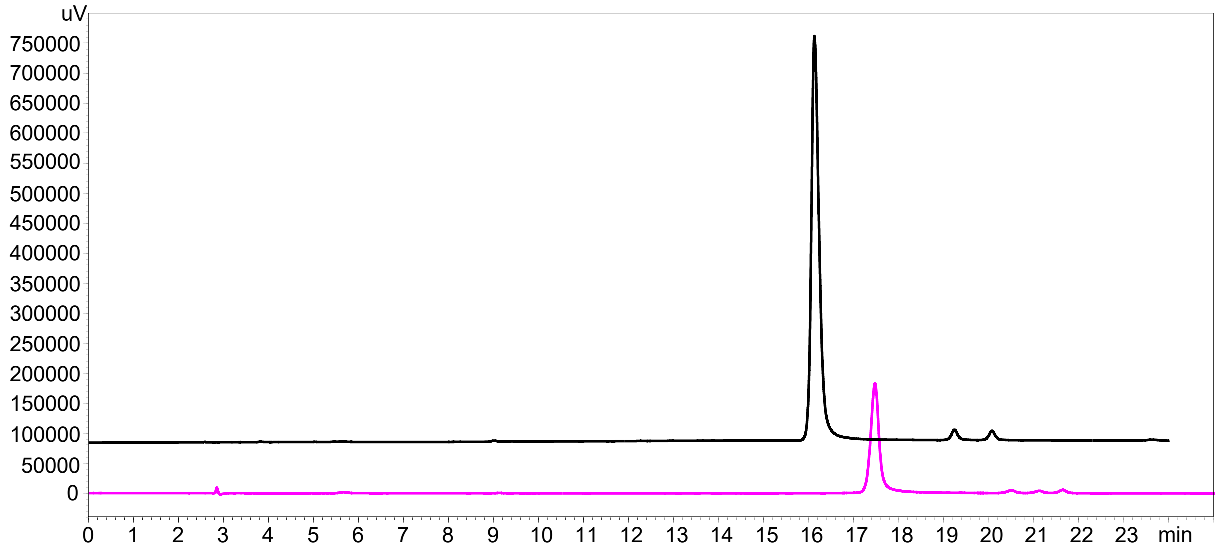

Figure S35: Comparison of HPLC chromatograms of **QUE** in various methods (λ_{max} = 360 nm). Method M1 in black, method M3 in pink

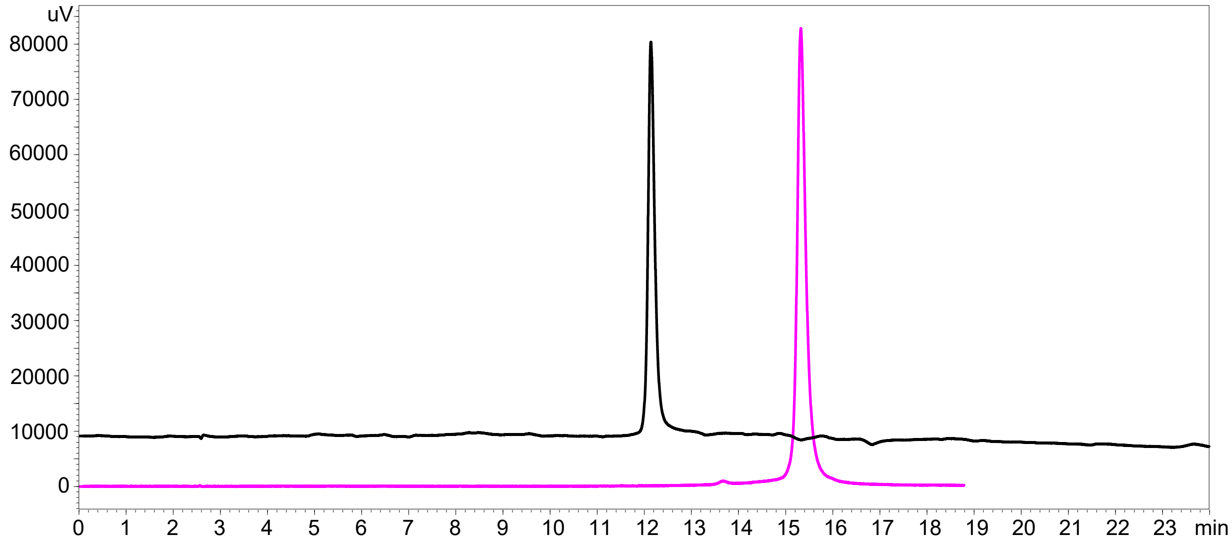

Figure S36: Comparison of HPLC chromatograms of **QUE-S** in various methods (λ_{max} = 360 nm). Method M1 in black, method M3 in pink

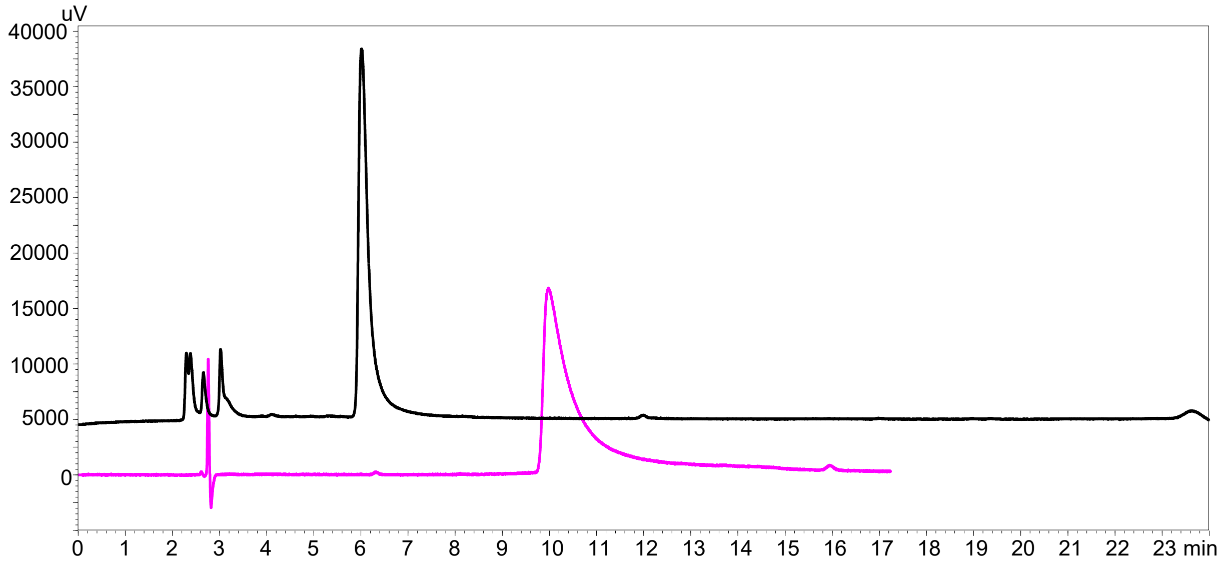

Figure S37: Comparison of HPLC chromatograms of **QUE-SS** in various methods (λ_{max} = 360 nm). Method M1 in black, method M3 in pink

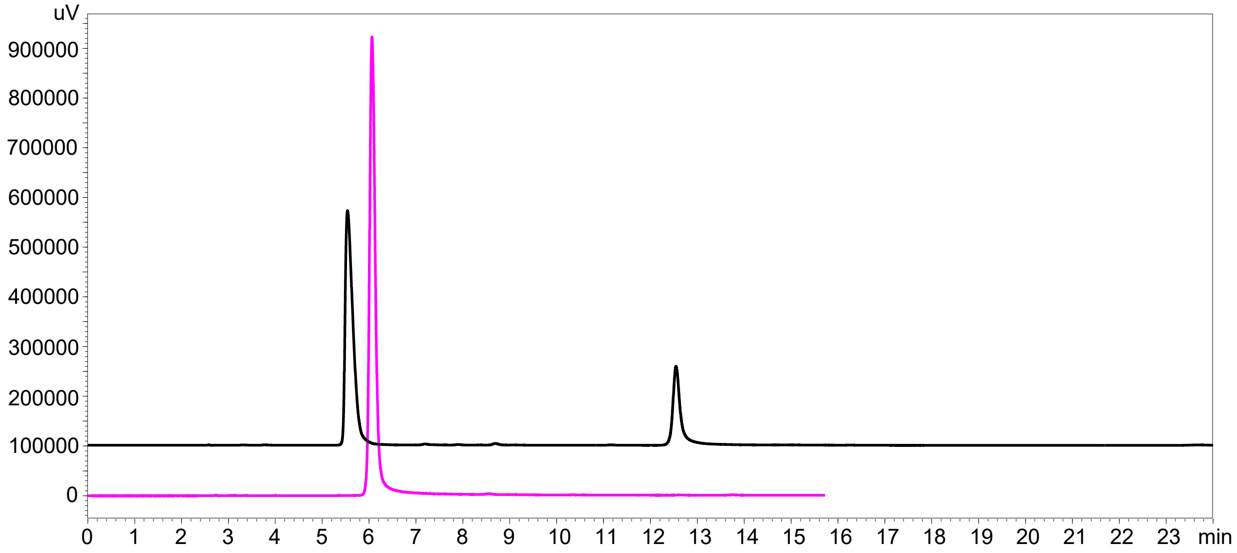

Figure S38: Comparison of HPLC chromatograms of **AMP** in various methods (λ_{max} = 285 nm). Method M1 in black, method M3 in pink

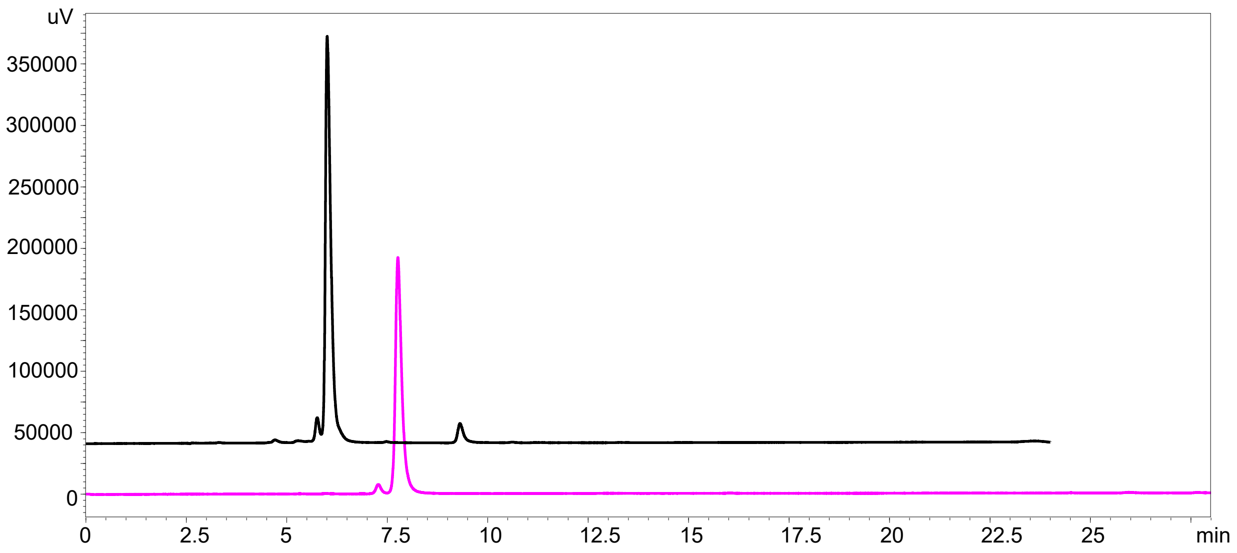

Figure S39: Comparison of HPLC chromatograms of **AMP-S** in various methods (λ_{max} = 285 nm). Method M1 in black, method M3 in pink

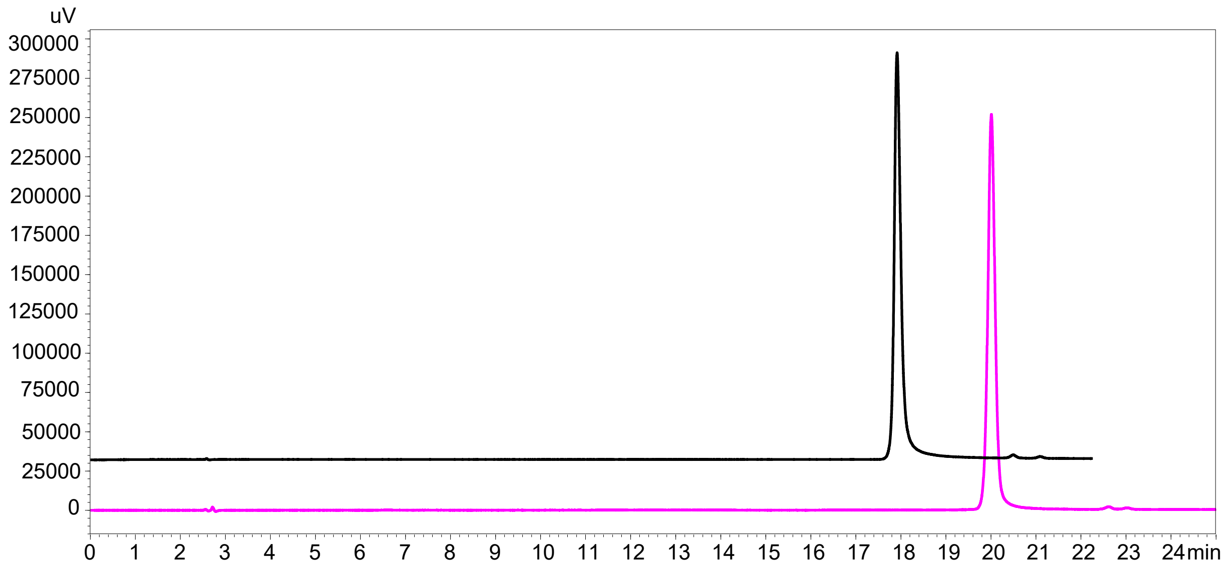

Figure S40: Comparison of HPLC chromatograms of **LUT** in various methods (λ_{max} = 285 nm). Method M1 in black, method M3 in pink

International Journal of
Molecular Sciences

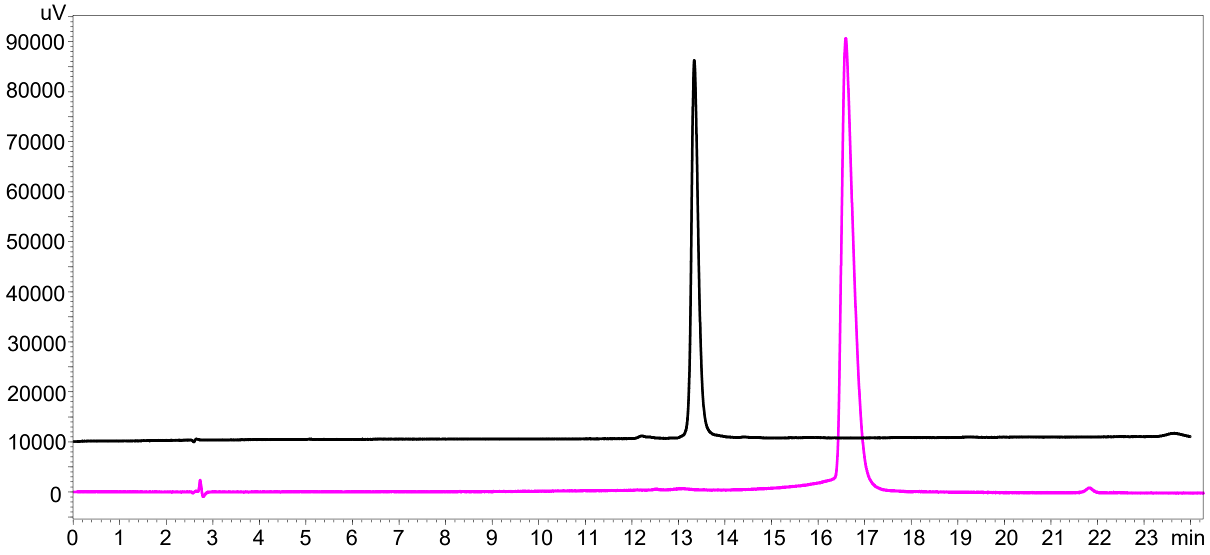

Figure S41: Comparison of HPLC chromatograms of **LUT-S** in various methods (λ_{max} = 338 nm). Method M1 in black, method M3 in pink

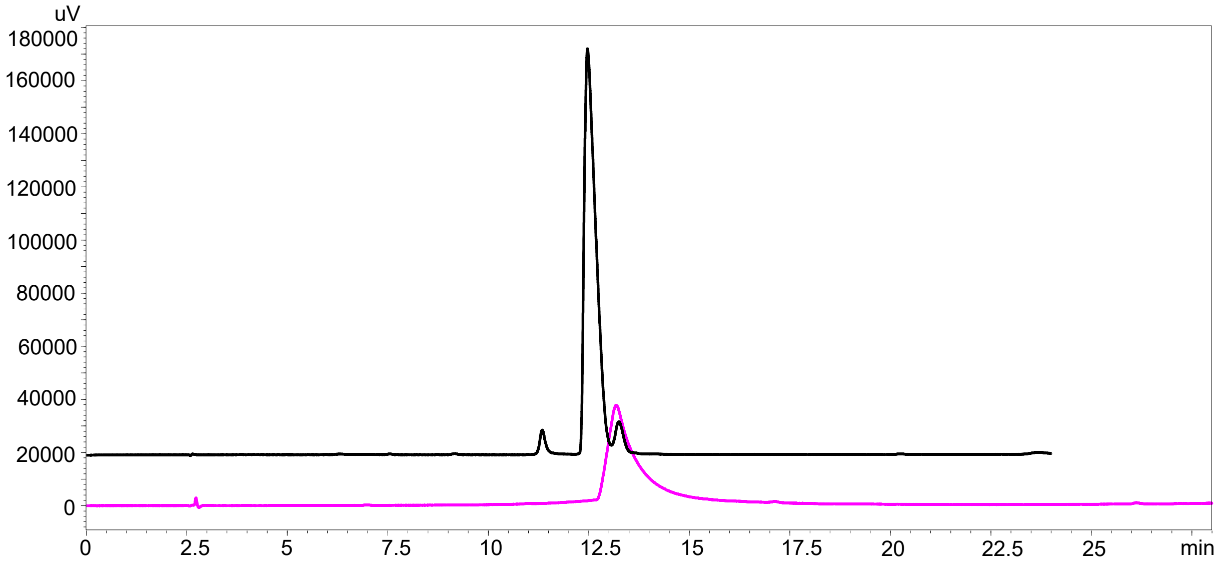

Figure S42: Comparison of HPLC chromatograms of **LUT-SS** in various methods (λ_{max} = 338 nm). Method M1 in black, method M3 in pink

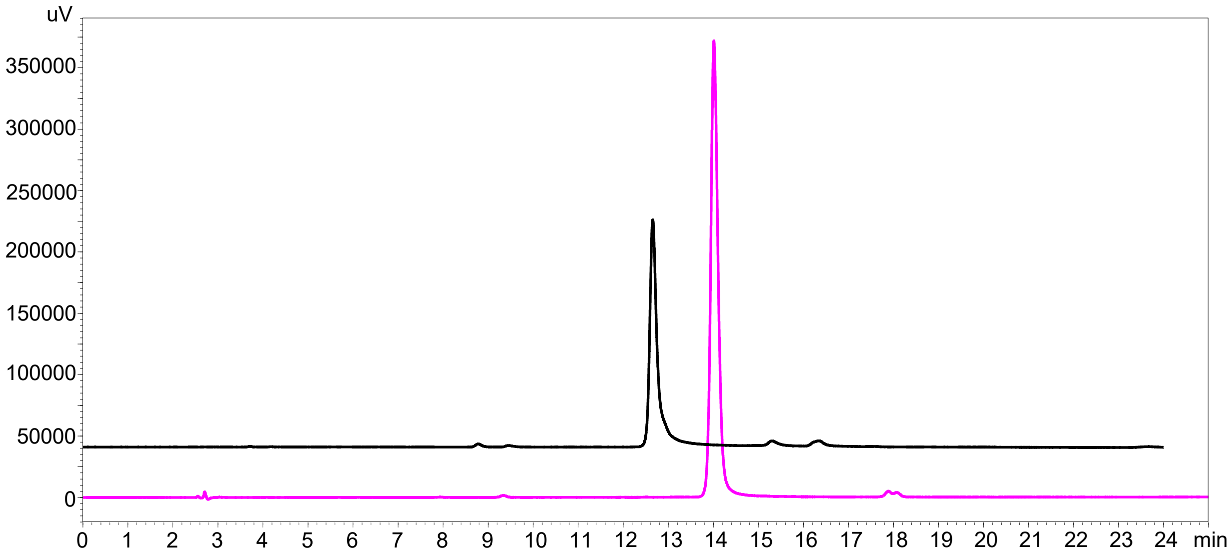

Figure S43: Comparison of HPLC chromatograms of **MYR** in various methods (λ_{max}= 365 nm). Method M1 in black, method M3 in pink

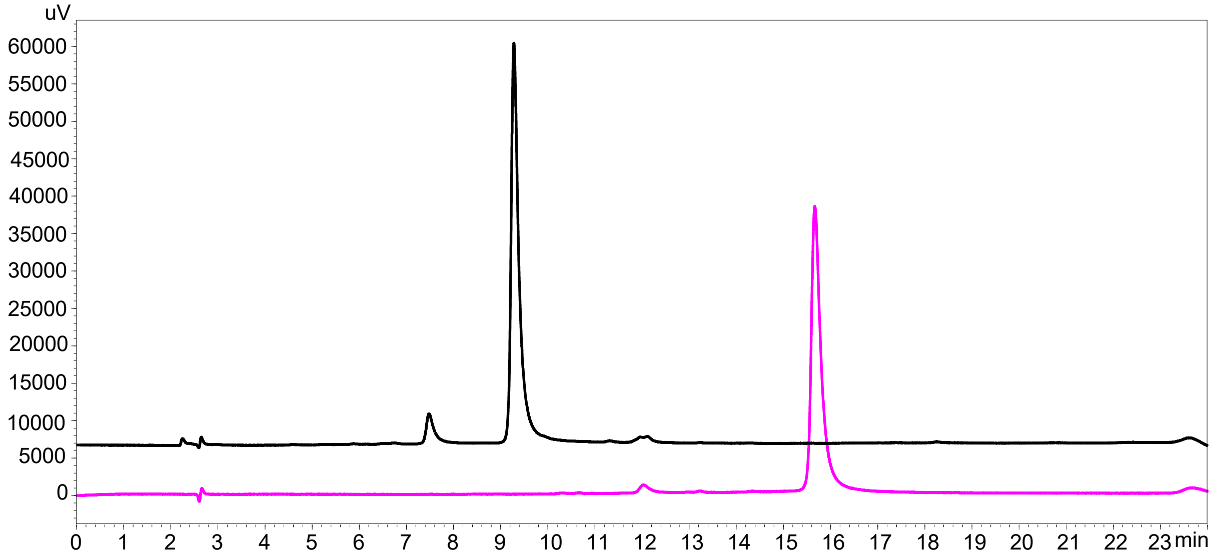

Figure S44: Comparison of HPLC chromatograms of **MYR-S** in various methods (λ_{max} = 365 nm). Method M1 in black, method M3 in pink

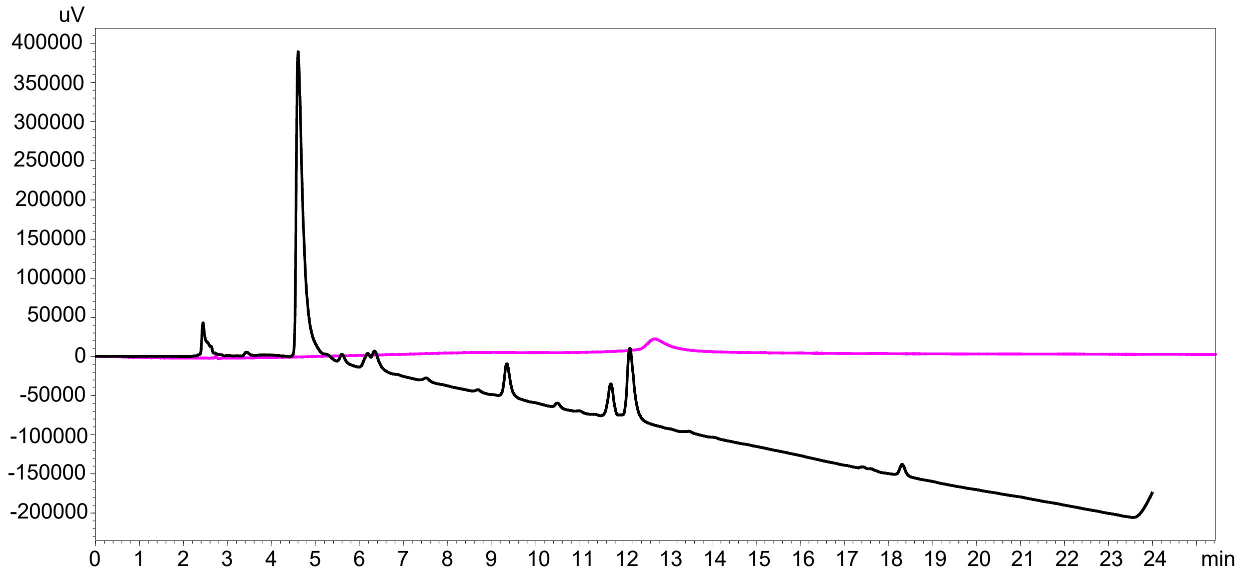

Figure S45: Comparison of HPLC chromatograms of **MYR-SS** in various methods (λ_{max} = 360 nm). Method M1 in black, method M3 in pink

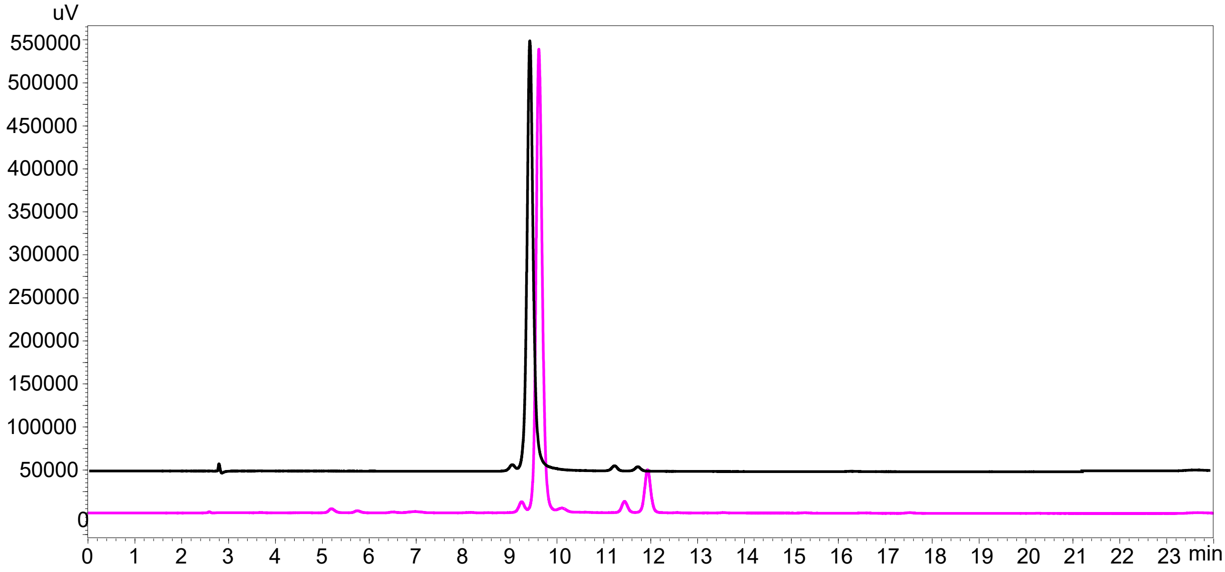

Figure S46: Comparison of HPLC chromatograms of **ISQ** in various methods (λ_{max} = 355 nm). Method M1 in black, method M3 in pink

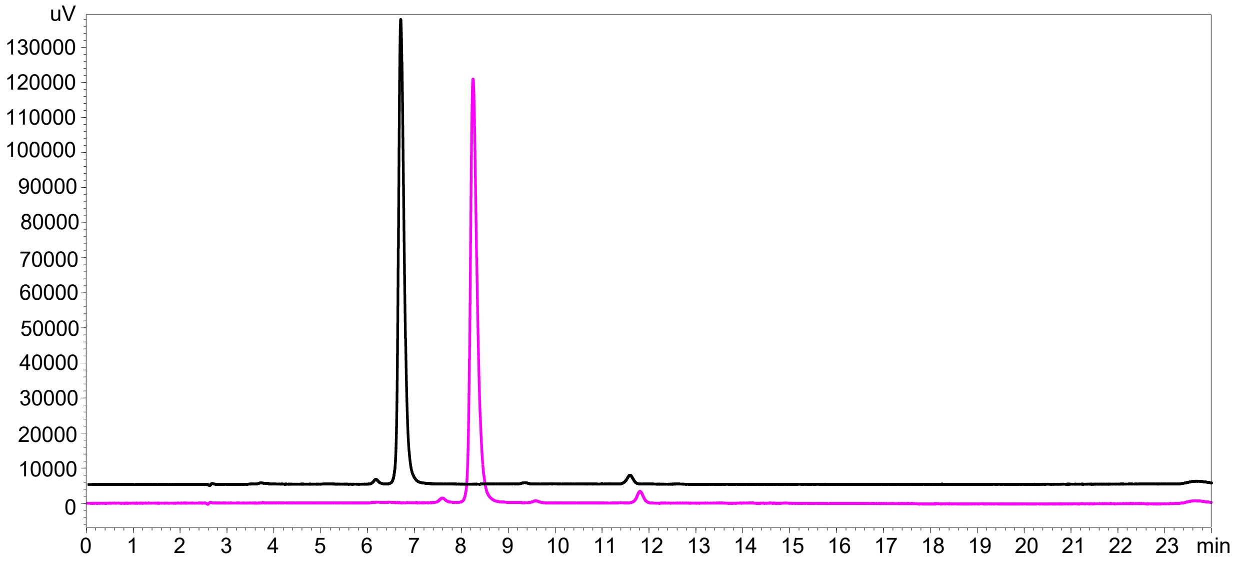

Figure S47: Comparison of HPLC chromatograms of **ISQ-S** in various methods (λ_{max} = 335 nm). Method M1 in black, method M3 in pink

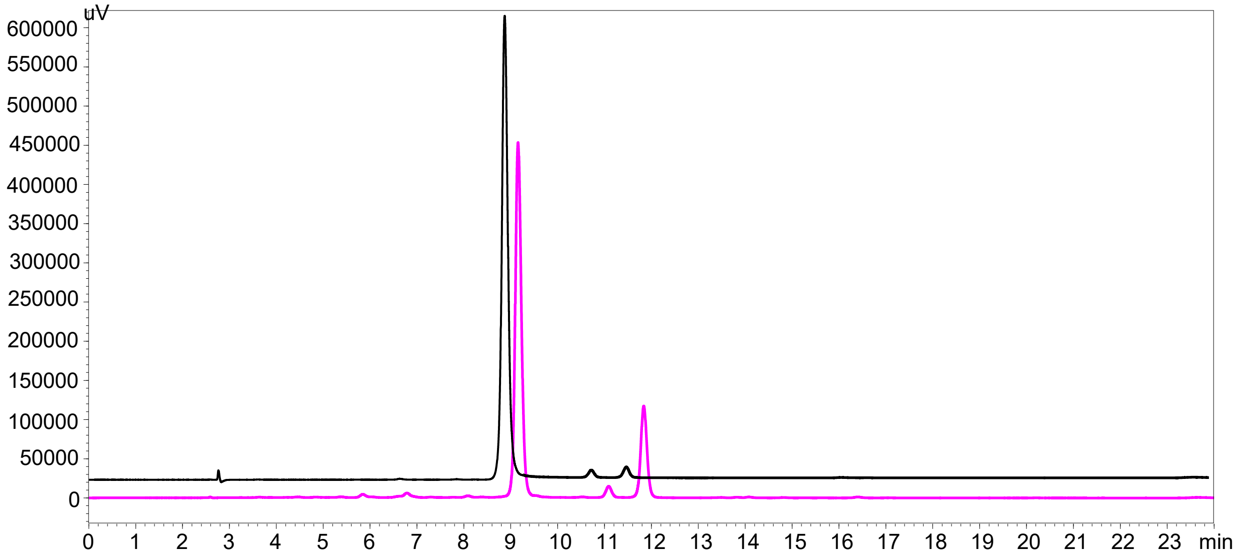

Figure S48: Comparison of HPLC chromatograms of **RUT** in various methods (λ_{max} = 355 nm). Method M1 in black, method M3 in pink

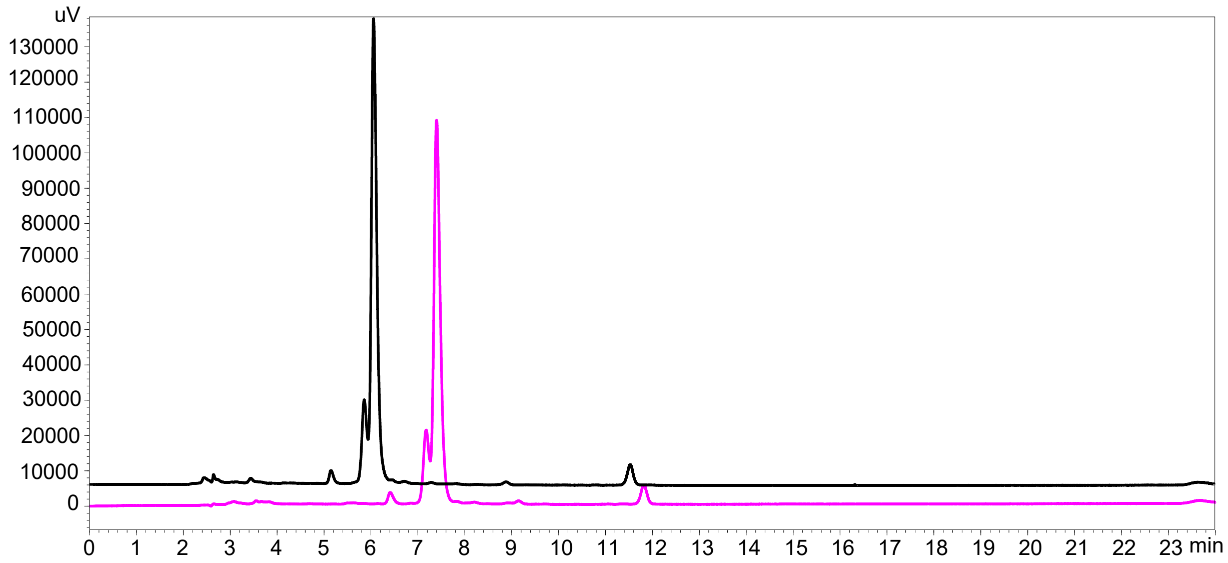

Figure S49: Comparison of HPLC chromatograms of **RUT-S** in various methods (λ_{max} = 355 nm). Method M1 in black, method M3 in pink

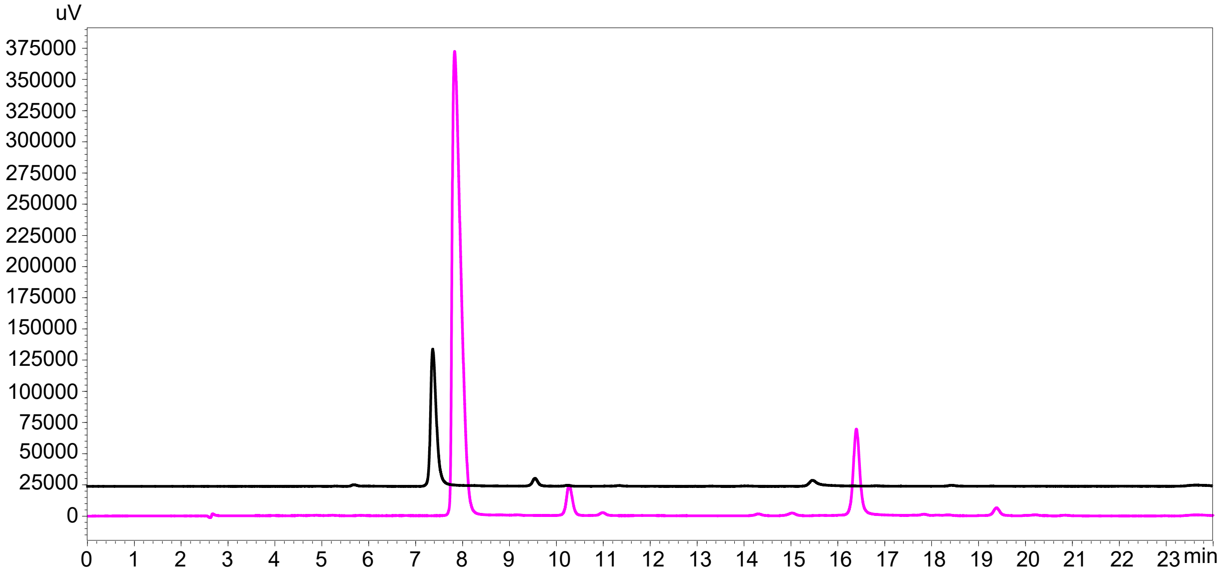

Figure S50: Comparison of HPLC chromatograms of **TAX** in various methods (λ_{max} = 285 nm). Method M1 in black, method M3 in pink

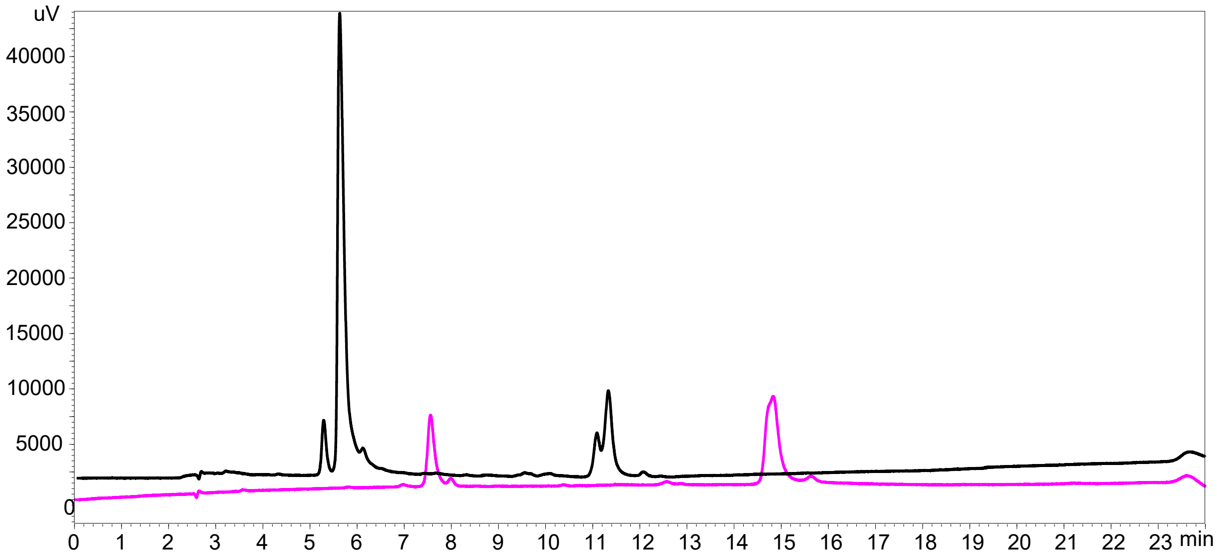

Figure S51: Comparison of HPLC chromatograms of **TAX-S** in various methods (λ_{max} = 285 nm). Method M1 in black, method M3 in pink

1. Purchartová, K.; Valentová, K.; Pelantová, H.; Marhol, P.; Cvačka, J.; Havlíček, L.; Křenková, A.; Vavříková, E.; Biedermann, D.; Chambers, C.S.; et al. Prokaryotic and eukaryotic aryl sulfotransferases: Sulfation of quercetin and its derivatives. *ChemCatChem* **2015**, *7*, 3152-3162, https://doi.org/10.1002/cctc.201500298.
